# Interplay between SMAD2 and STAT5A is a critical determinant of IL-17A/IL-17F differential expression

**DOI:** 10.1186/s43556-021-00034-3

**Published:** 2021-04-01

**Authors:** Karla Fabiola Corral-Jara, Camille Chauvin, Wassim Abou-Jaoudé, Maximilien Grandclaudon, Aurélien Naldi, Vassili Soumelis, Denis Thieffry

**Affiliations:** 1 Computational Systems Biology Team, Institut de Biologie de l’École Normale Supérieure, CNRS UMR8197, INSERM U1024, École Normale Supérieure, PSL Université, 75005 Paris, France; 2 Integrative Biology of Human Dendritic Cells and T Cells Team, Institut de Recherche St-Louis, U976, Hôpital Saint Louis, 75010 Paris, France; 3 Institut Curie, Centre de Recherche, PSL Research University, 75005 Paris, France; INSERM U932, Immunity and Cancer, 75005 Paris, France

**Keywords:** Th17 differentiation, IL-17A, IL-17-F, STAT5, SMAD2, Systems biology, Logical modeling

## Abstract

Interleukins (IL)-17A and F are critical cytokines in anti-microbial immunity but also contribute to auto-immune pathologies. Recent evidence suggests that they may be differentially produced by T-helper (Th) cells, but the underlying mechanisms remain unknown. To address this question, we built a regulatory graph integrating all reported upstream regulators of IL-17A and F, completed by ChIP-seq data analyses. The resulting regulatory graph encompasses 82 components and 136 regulatory links. The graph was then supplemented by logical rules calibrated with original flow cytometry data using naive CD4^+^ T cells, in conditions inducing IL-17A or IL-17F. The model displays specific stable states corresponding to virtual phenotypes explaining IL-17A and IL-17F differential regulation across eight cytokine stimulatory conditions. Our model analysis points to the transcription factors NFAT2A, STAT5A and SMAD2 as key regulators of the differential expression of IL-17A and IL-17F, with STAT5A controlling IL-17F expression, and an interplay of NFAT2A, STAT5A and SMAD2 controlling IL-17A expression. We experimentally observed that the production of IL-17A was correlated with an increase of SMAD2 transcription, and the expression of IL-17F correlated with an increase of BLIMP-1 transcription, together with an increase of STAT5A expression (mRNA), as predicted by our model. Interestingly, RORγt presumably plays a more determinant role in IL-17A expression as compared to IL-17F expression. In conclusion, we propose the first mechanistic model accounting for the differential expression of IL-17A and F in Th cells, providing a basis to design novel therapeutic interventions in auto-immune and inflammatory diseases.

**Supplementary Information:**

The online version contains supplementary material available at 10.1186/s43556-021-00034-3.

## Introduction

Cluster of differentiation (CD)4^+^ T helper (Th) cells play critical roles in orchestrating adaptive immune responses. Th cell differentiation relies on a complex network involving specific input interleukins (IL) and other cytokines, signaling pathways, and transcription factors (TF), which together specify the phenotype acquired by naive CD4^+^ T cells upon activation [1].

Among all CD4^+^ T cells, Th17 cells have been extensively described as crucial in the host defense against microbes, including bacteria and fungi [2], and as pathogenic in a broad spectrum of inflammatory and autoimmune diseases [3]. Human Th17 differentiation results from the synergistic integration of four different cytokines: IL-1β, IL-6, IL-23 and Ttransforming growth factor beta (TGF-β) [4, 5]. Although IL-22, TNF-α and C-C chemokine receptor type 6 (CCR6) have been associated with Th17 phenotype [6], the best phenotypical markers describing this Th subset are IL-17A and IL-17F, which were extensively associated with Th17 function in health and disease [7].

IL-17A and IL-17F were first assumed to function redundantly, because they share 50% of homology in amino-acid sequence [8], induce similar pro-inflammatory cytokines [9] and bind the same IL-17RA/IL-17RC heterodimeric receptor [10, 11]. However, recent evidence suggests that these two cytokines play non-overlapping and even opposite roles [12]. First, it has been recently reported that IL-17F binds IL-17RC homodimers, while IL-17A does not, suggesting that IL-17F may trigger distinct cellular functions, as compared to IL-17A [13, 14]. Furthermore, several disease models indicate that IL-17A and F may have distinct functions. In an experimental allergic encephalomyelitis (EAE) model, only *Il17a−/−* mice showed a significantly reduced disease score, indicating a requirement for IL-17A but not for IL-17F in the initiation of EAE [15]. In contrast, *Il17f−/−* animals exhibit higher Th2 cytokine and eosinophil infiltration in an asthma model, suggesting a suppressive function for IL-17F cytokine [15]. In addition, it has been recently shown that absence of IL-17F but not of IL-17A could protect mice against colitis [16].

Interestingly, several studies suggest that IL-17A and F could be differentially regulated under specific conditions. First, IL-17A promoter, but not IL-17F promoter, displays conserved calcium-related nuclear factor of activated T cells (NFAT) binding sites bound by NFATc1 (NFAT2) [17, 18]. Second, the protein kinase C alpha (PKCα) directly regulates the kinase activity of TGFBR1, which itself activates members of SMAD TF family, and PKCα maintains an effective IL-17A response, but not IL-17F expression [19]. Finally, we recently showed that IL-12 induces the production of IL-17F but not of IL-17A, in presence of IL-1β, during Th differentiation. This brought to light a positive role of IL-12 in Th17 differentiation, which remains without mechanistic explanation [20].

These elements suggest that IL-17A and F may be produced together but also separately, thereby defining subpopulations of Th17 cells playing distinct physiopathological roles, involving distinct regulatory mechanisms during Th differentiation.

**Fig. 1:**
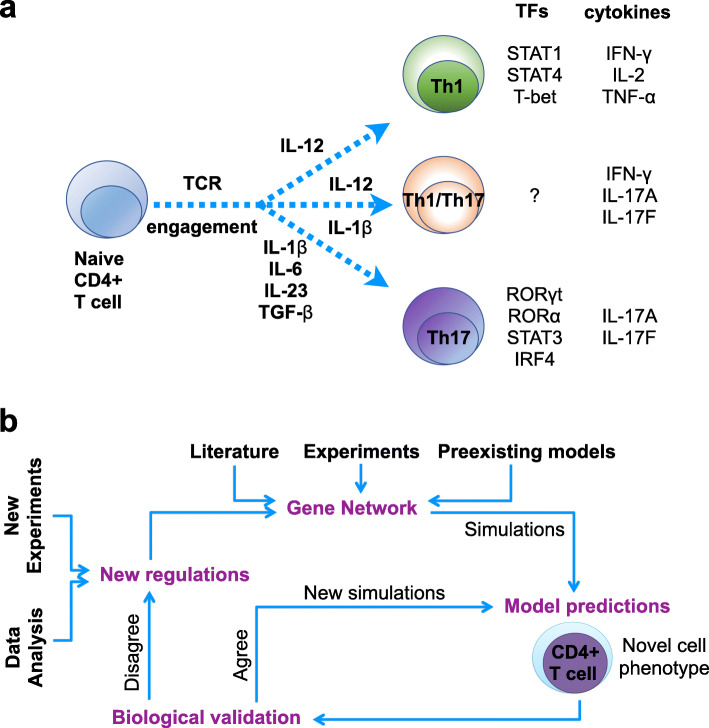
Schematic overview of the experimental and computational workflow. **a** Schematic representation of Th1, Th17 and hybrid Th1-Th17 cell specification. TCR engagement, input cytokines, transcription factors and output cytokines are considered in a Th differentiation event. **b** Iterative modeling workflow. A model is first built based on information collected from publications, databases and previous models. This model is then used to predict dynamical behaviors (cell phenotype, differentiation, reprogramming, and so forth). Predictions are compared with experimental data; when the predictions and experimental data agree, further predictive simulations are performed; when they do not agree, further regulations are inferred from ChIP-seq and RNA-seq data, and are integrated into the model until simulations fully agree with biological data. TCR: T-cell receptor; TF: Transcription Factor

In this study, we sought to decipher the mechanisms underlying the observed differential expression of IL-17A and IL-17F observed in human Th cells. Specifically, we addressed the intracellular mechanisms underlying the positive role of IL-12 in IL-17F regulation. In this respect, we developed a dynamical network model focused on IL-17A and F regulation (Fig. 1). Our model analysis showed that the activation levels of the TF SMAD2, Signal transducer and activator of transcription (STAT) 5A, and NFAT2A influence IL-17A expression in distinct proTh17 conditions, including IL-12 signaling. Thereby, we provide a systems level explanation for the observed uncoupling between IL-17F and IL-17A expression. Our model analysis further identified specific regulatory mechanisms and potential targets for pharmacological manipulations.

## Results

### Delineation of the regulatory graph controlling Th1-Th17 cell differentiation

We first conducted an extensive analysis of published data to identify the key signaling events and molecules driving Th17 differentiation upon exposure to antigen and various cytokine combinations, including IL-1β, IL-23, IL-6 and TGF-β. We also curated the literature for the IL-12 pathway in T cells in order to understand its interplay with known Th17 inducers.

The construction of the regulatory graph relied on 91 original articles, two KEGG pathways (hsa04350, hsa04660, https://www.genome.jp/kegg/), the T-MOD database [20], a Th17 differentiation data-driven model [21], and two preexisting Th differentiation dynamical models [22, 23]. We also used public ChIP-seq data to further validate several putative interactions. Noteworthy, we thereby detected IL-17A and IL-17F genomic areas enriched for STAT1 and STAT5 binding sites (Supplementary Fig. 1; Supplementary Table 1 lists the ChIP-seq data sources).

**Fig. 2:**
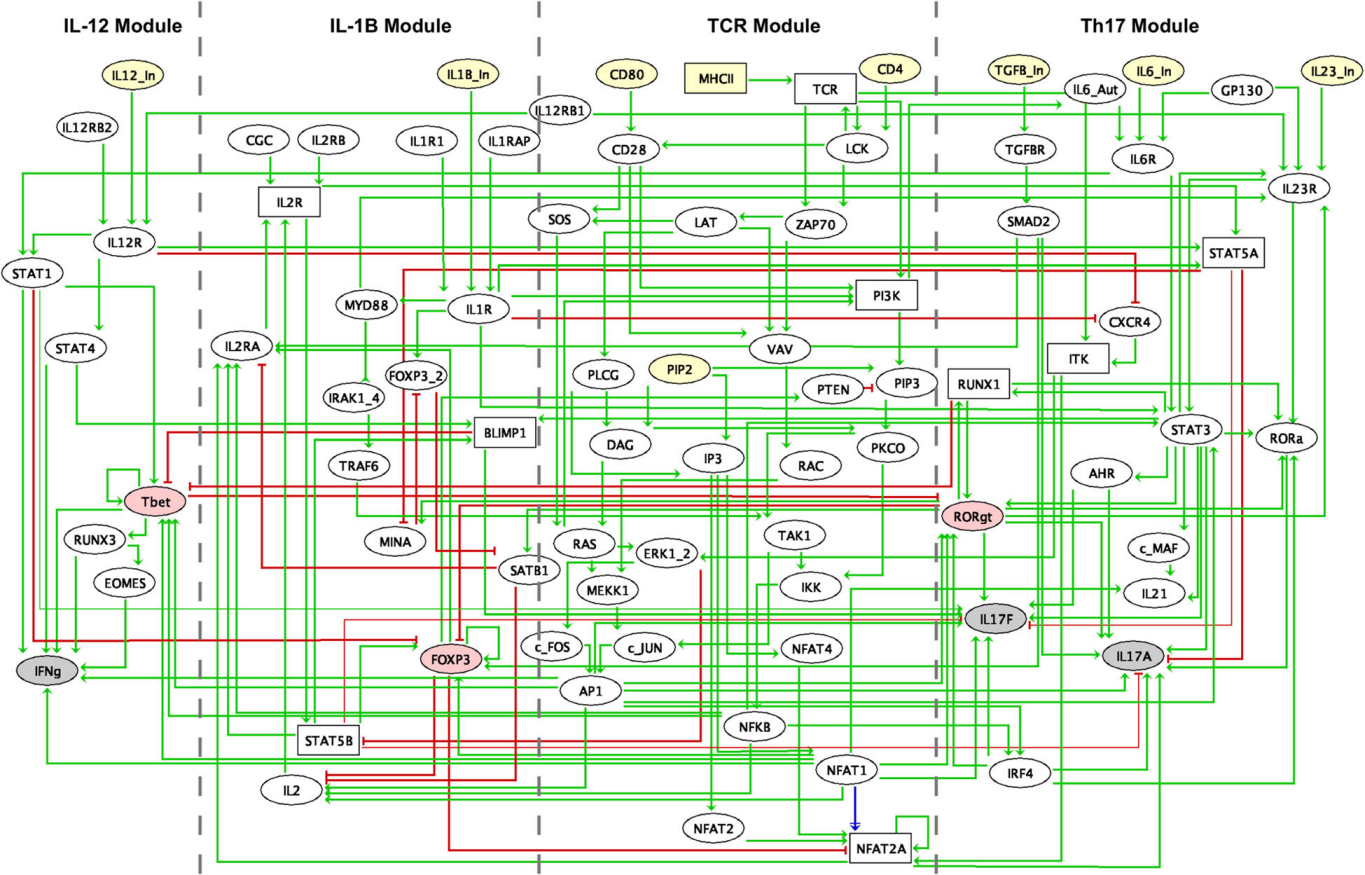
Regulatory graph integrating the interactions inferred from the literature, databases and ChIP-seq data meta-analyses. Four CD4^+^ T cell differentiation modules are considered to construct the regulatory graph: Th1, IL-1β, TCR and Th17 modules. Nodes represent genes and arrows denote regulatory interactions. Yellow nodes denote inputs, pink nodes denote master transcription factors, and gray nodes denote outputs. Ellipses represent Boolean components, while rectangles represent the ternary components. Green and red edges correspond to activations and inhibitions, respectively. Further information about each node, including supporting references, can be found in Supplementary Table 4

All this information has been integrated into a regulatory graph **(**Fig. 2**)**, encompassing 82 nodes, including nine input components and four key output components (Interferon (IFN)-γ, IL-17A, IL-17F, IL-21). The graph encompasses 120 positive, fifteen negative and one dual interactions.

The model captures critical signaling events involved in Th1 and Th17 cell differentiation, from stimulatory signals by cell surface receptors to the activation of TFs and the production of critical proteins. Our model encompasses four distinct modules: the T cell receptor (TCR) central module, the Th17 module, the IL-1β module and the IL-12 module.

The TCR central module encompasses the three signals involved in the activation of naive T cells: (i) the major histocompatibility complex II (MHC II)/peptide complexes on the antigen presenting cells (APC), (ii) the binding of the costimulatory ligands CD80 or CD86 on the APC to CD28 on the T cell, and (iii) CD4 interactions, a cell surface glycoprotein that binds to a monomorphic region of MHC class II molecules, and thereby stabilizes the interaction between the TCR and MHC class II. The combined signals from the TCR and CD28 lead to the activation of NFAT, activator protein 1 (AP-1), and nuclear factor kappa B (NF-kB). These TFs promote the expression of IL-2 and the gene encoding a subunit of IL-2 receptor (IL-2R) (IL-2RA, also called CD25). The β and γ subunits of IL-2R are constitutively present. Binding of IL-2 to IL-2R results in the activation of STAT5A and STAT5B. STAT5A in turn activates the forkhead box p3 (FOXP3) TF, expressed mainly in regulatory T cells, while STAT5B further modulates the expression of the TF B lymphocyte-induced maturation protein-1 (BLIMP-1). BLIMP-1 activates IL-17F, but not IL-17A, and inhibits the expression of the TF T-box expressed in T cells (T-bet) (TCR module, Fig. 2).

The Th17 module encompasses the input cytokines TGF-β, IL-6, IL-23 and IL-1β, which induce a Th17 phenotype (proTh17 cytokines). IL-6, IL-23, and IL-1β signaling activates STAT3, which in turn leads to RAR-related orphan receptor gamma (RORγt, encoded by RORC gene) activation. TGF-β induces the expression of SMAD2, which is a direct positive regulator of IL-17A expression (Th17 module, Fig. 2).

IL-1β signaling has an inherent relationship with the TCR module, as it is able to activate phosphoinositide 3-kinases (PI3K) and to induce myeloid differentiation primary response 88 (Myd88) expression, which results in AP-1 activation (IL-1β module, Fig. 2).

The IL-12 module includes the input cytokine IL-12, which can trigger the activation of both STAT1 and STAT4, leading to the expression of the master transcriptional regulator T-bet and to the production of IFN-γ cytokine (IL-12 module, Fig. 2). It is usually considered that Th1 and Th17 differentiation programs are mutually exclusive, with mutual inhibitions between T-bet and RORγt. However, based on our literature search, we were led to consider additional cross-talks, in particular an activation of IL-17F by STAT1 and an activation of STAT1 by IL6R.

### Delineation of multilevel components

The activity of most regulatory components can be represented by a Boolean variable, taking the value 0 (OFF) when the activity level of the component is negligible, or 1 (ON) when the activity level of the component is sufficiently high to enable a regulatory effect. However, when justified, multilevel variables can be used to account for more subtle situations. In particular, we considered three possible activity levels (0, 1 and 2) for the T cell receptor (TCR), corresponding to negligible, moderate, and high levels, in order to more accurately represent the effects of varying antigen concentrations on the receptor. We also associated a ternary variable with MHC II, with its highest level (value 2) required to trigger TCR at its highest level (value 2).

Similarly, we associated a ternary variable with PI3K, mirroring the different levels of the TCR, with the highest level of PI3K required to activate phosphatidylinositol (3,4,5)-trisphosphate (PIP3). STAT5A and STAT5B were also associated with ternary variables, to encode differences in the relative amounts and effects of these proteins.

For each ternary component, we further defined the thresholds of the outgoing interactions in order to match the observed differences in the effects of moderate versus high doses. We assumed that the inhibitions of IL-17A and IL-17F by STAT5A and STAT5B require high activation levels (value 2) of these STATs, while the activations of IL-2RA and BLIMP-1 require only a moderate level (value 1) of STAT5B, to indicate that activation effects of STATs on their targets can be achieved with a moderate level, whereas the strong inhibition effects of STATs on IL-17 require a high level.

**Fig. 3:**
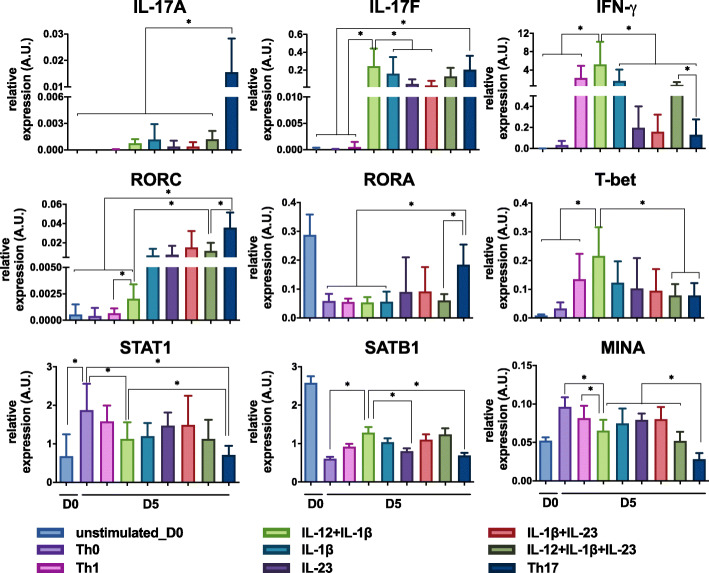
Quantification of transcription of model internal components by RT-PCR. Human naive T cells were differentiated for five days in the presence of polyclonal activation (anti-CD3/anti-CD28 beads). Cells were cultured in the presence of different cytokine inputs: proTh1 (IL-12), IL-1β, IL-23, IL-12 + IL-1β, IL-1β + IL-23, IL-12 + IL-1β + IL-23, proTh17 (IL-1β + IL-6 + IL-23 + TGF-β). Cells exposed only to polyclonal stimulation were considered as Th0. RNA extraction of differentiated cells was performed and transcripts were then quantified by RT-PCR. Gene expression was normalized to the housekeeping genes HPRT1, B2M and RPL34. Relative expression of IL-17A, IL-17F, IFN-γ, RORC, RORA, T-bet, STAT1, SATB1, MINA are depicted. Graphs represent mean ± SD, *N* = 5 and * denotes *p* < 0.05 (Wilcoxon test)

Gene expression of some internal model components was assessed by quantitative reverse transcription polymerase chain reaction (RT-PCR), for diverse input conditions (Th0, proTh1 (IL-12), IL-1β, IL-23, IL-12 + IL-1β, IL-1β + IL-23, IL-12 + IL-1β + IL-23, proTh17 (IL-1β + IL-6 + IL-23 + TGF-β)) to complement model construction and delineation of multilevel components. IL-17A and IL-17F were detected together only in the Th17 condition, while IL-17F and IFN-γ were detected in the IL-12 + IL-1β condition. RORC and RORA transcription was minor in IL-12 + IL-1β-differentiated cells as compared to the Th17 cells. Special AT-rich sequence-binding protein 1 (SATB1) and MYC induced nuclear antigen (MINA) transcription significantly decreased in the Th17 conditions. STAT1 transcription was decreased in the IL-12 + IL-1β and the Th17 conditions as compared to the IL-12 condition (Fig. 3). In contrast, the transcription of STAT3 and SMAD2 does not seem to vary strongly between the IL-12 + IL-1β and proTh17 conditions (Supplementary Fig. 2a, b). In addition, the expression of C-X-C motif chemokine receptor 4 (CXCR4) was up-regulated in Th17-polarized cells, as shown by flow cytometry (Supplementary Fig. 2c).

### Th1-Th17 model calibration and validation

**Fig. 4:**
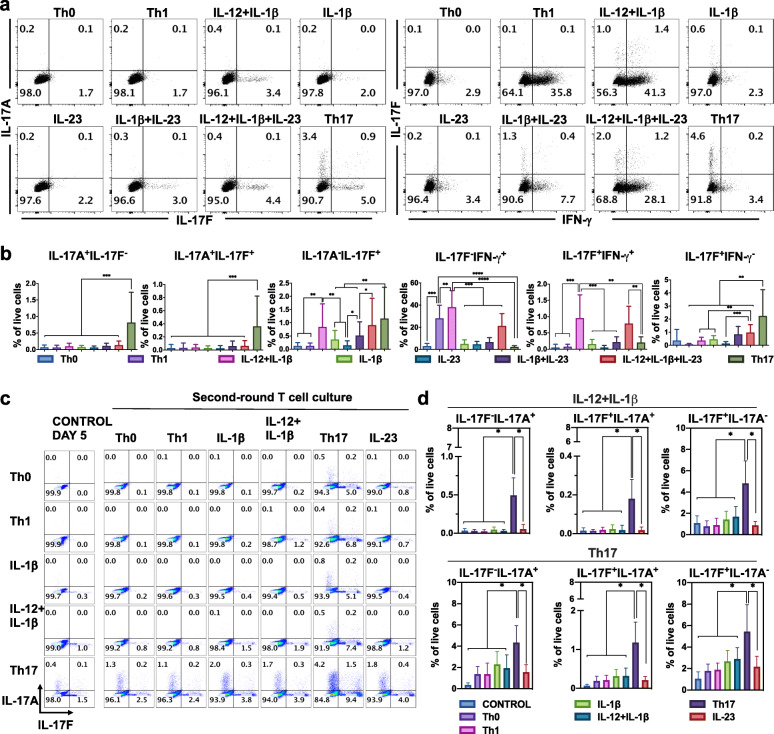
Experimental validation of CD4^+^ T cell phenotypes. Human naive T cells were differentiated for 5 days in the presence of polyclonal activation (anti-CD3/anti-CD28 beads). Cells were cultured in the presence of different cytokine inputs: proTh1 (IL-12), IL-1β, IL-23, IL-12 + IL-1β, IL-1β + IL-23, IL-12 + IL-1β + IL-23, proTh17 (IL-1β + IL-6 + IL-23 + TGF-β). Cells exposed only to polyclonal stimulation were considered as Th0. Differentiated cells were then subjected to flow cytometry analysis. **a** Dot plots representing the IL-17A and IL-17F cells in live CD4^+^ cells are shown on the left. Dot plots representing the IL-17F and IFN-γ cells in live CD4^+^ cells are shown on the right. Representative data from three independent experiments are shown. Numbers denotes frequency of gated cells. **b** The frequency of cells for each subset in Apannel a is shown. Graphs represent mean ± SD, *N* = 12, *, ** and *** denotes *p* < 0.05, *p* < 0.01 and *p* < 0.001, respectively (Wilcoxon test). **c** Cells were submitted to a second round of polarization in the presence of the cytokine inputs: IL-1β, IL-12, IL-23, IL-12 + IL-1β, proTh17, for two additional days. Dot plots representing the IL-17A and IL-17F cells in live CD4^+^ cells and frequency of cells are shown. **d** The frequency of cells for each subset in Cpannel c is shown. Graphs represent mean ± SD, *N* = 6, * represents *p* < 0.05 (Wilcoxon test)

In order to calibrate our logical model, we conducted a series of de novo flow cytometry experiments on naive T cells in a selection of cytokine conditions of interest. Human naive T cells were differentiated for 5five days in the presence of polyclonal activation (anti-CD3/anti-CD28 beads) to mimic antigen stimulation. Cells were cultured in the presence of different cytokine inputs to achieve a wide variety of polarization profiles (Fig. 4a, b): proTh1 (IL-12), IL-1β, IL-23, IL-12 + IL-1β, IL-1β + IL-23, IL-12 + IL-1β + IL-23, proTh17 (IL-1β + IL-6 + IL-23 + TGF-β). Cells exposed only to polyclonal stimulation and no input cytokines were considered as Th0. IL-17A production was observed only in the proTh17 condition, while IL-17F production appeared at various levels in several input conditions (IL-1β, IL-12 + IL-1β, IL-12 + IL-1β + IL-23, and proTh17). Furthermore, in proTh17 condition, three distinct phenotypes were observed with a fraction of cells producing only IL-17F, another producing only IL-17A, and another one producing both cytokines, as previously shown [20]. The proportion of IL-17F^+^ cells tended to increase in IL-12 + IL-1β + IL-23 condition. IL-17F^+^IFN-γ^+^ hybrid cells were only observed in the IL-12 + IL-1β and IL-12 + IL-1β + IL-23 conditions, but not in the other six conditions tested. Of note, the addition of IL-12 after 3three days of culture in IL-1β + IL-23 condition did not impact the proportion of IL-17F-^-^ and IFN-γ-^-^producing cells (Supplementary Fig. 3a, b).

Based on these results, we tuned the logical rules of the model to reproduce the observed polarizing events in the different cytokine environments (see Supplementary Table 2 for a description of the input combinations tested in the model). Firstly, we performed a stable state analysis of the model for the eight input conditions considered. After several rounds of calibration, our model was able to capture the phenotypes obtained in each condition in terms of a logical stable state. Altogether, a total of 103 stable states were identified distributed across input conditions as follow: No cytokine inputs (10), IL-12 (7), IL-1β (14), IL-23 (16), IL-12 + IL-1β (14), IL-1β + IL-23 (14), IL-12 + IL-1β + IL-23 (14), Th17 (14).

As a mean to validate the model, we assessed by flow cytometry the stability of the cell phenotypes producing IL-17A or IL-17F. To do so, the different cell subtypes were submitted to a second round of polarization in the following culture conditions: medium, IL-1β, IL-12, IL-23, IL-12 + IL-1β, proTh17, for two additional days. As depicted in Fig. 4c-d, the cells primarily polarized with IL-12 + IL-1β retained their capability to produce IL-17F in the different conditions. These cells further acquired the capability to produce minor quantities of IL-17A when exposed to the proTh17 cocktail, but this secretion was weaker than that achieved by the cells previously polarized with the proTh17 cocktail.

**Fig. 5:**
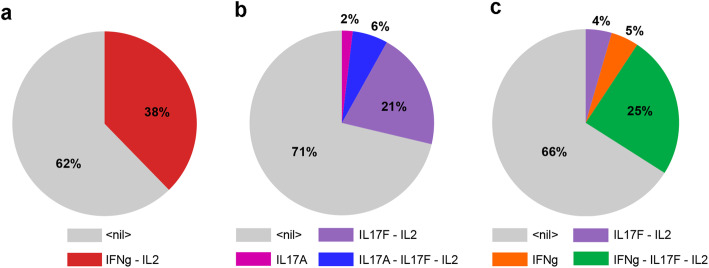
Stochastic simulations of the wild type model. MaBoSS was used to simulate the evolution of cell populations for each of the considered environmental conditions, starting from naive Th0 state. The probabilities associated with each phenotype is computed from the number of stochastic simulations leading to each phenotype from pre-defined initial conditions. **a** Wild type simulation in the presence of pro-Th1 cytokines, which gives rise to IFN-γ^ +^ cells. **b** Wild type simulation in the presence of pro-Th17 cytokines, which gives rise predominantly to IL-17F+^+^ cells, a smaller proportion of double IL-17A +^+^ IL-17F+^+^, and a still smaller proportion of single IL-17A+^+^ cells. **c** Wild type simulation in the presence of IL-12 + IL-1β, which give rise to cells expressing combination of IL-2, IFN-γ and/or IL-17F (but not IL-17A). In each case, the large grey sector correspond to cells remaining in a non activated state. Although the percentages obtained are sensitive to updating rates, these simulations point to key differences in the specific cell phenotypes obtained for different combinations of Th1/Th17 polarizing cytokines

In order to check whether the observed phenotypes could be reached in the corresponding input condition, starting from Th0 conditions, we performed stochastic simulations using the software MaBoSS (see Material and Methods) to recapitulate the differentiation of Th1, Th17, Th1-Th17 hybrid cells. After tuning several logical rules, our model was able to qualitatively reproduce the polarizing events observed experimentally. Firstly, we could recover the polarization towards Th1 and Th17 cell types, characterized by the expression of IFN-γ and IL-17A/IL-17F, respectively, under the corresponding polarizing cytokine environments (Fig. 5a and b) (Supplementary Table 3, top). We then verified that IL-17F^+^IFN-γ^+^IL-17A^−^ and IL-17F^+^ cell phenotypes could be induced in the IL-12 + IL-1β condition (Fig. 5c) (Supplementary Table 3, bottom). We also captured the phenotypes expressing IL-17A alone, IL-17F alone, and both IL-17A and IL-17F, induced experimentally in proTh17 conditions (Supplementary Table 3, bottom). Our model simulations could also reproduce the experimental results obtained after re-stimulation (Fig. 4c and d), showing in particular that cells primarily polarized with IL-12 + IL-1β can produce IL-17A when exposed to proTh17 input conditions.

The delineation of most logical rules was straightforward, excepting some specific cases requiring further tuning to match the experimental observations, in particular IL-17A and IL-17F. Supplementary Table 4 lists the components of the resulting model, with the corresponding logical rules, together with supporting references.

### Identification of key internal model components underlying IL-17A and IL-17F differential expression

To identify candidate components putatively involved in IL-17A versus IL-17F differential expressions in Th cells, we performed a comparative analysis of model component levels in IL-17A^+^, IL-17F^+^ and IL-17A^+^IL-17F^+^ phenotypes, as obtained in IL-12 + IL-1β and proTh17 condition (IL-1β + IL-6 + IL-23 + TGF-β) using stochastic simulations with MaBoSS (see Material and Methods).

More specifically, we compared four virtual phenotypes obtained in the following conditions: (i) IL-17F^+^ and IFN-γ^+^IL-17F^+^ cell phenotypes observed in IL-12 + IL-1β condition, and (ii) IL-17A^+^ and IL-17A^+^IL-17F^+^ cell phenotypes achieved in proTh17 condition.

Our analysis delineated nineteen components (Supplementary Fig. 4) that reached different activation values across the considered phenotypes. We found that IL-12R, STAT4, NFAT2A, STAT5A, T-bet, RUNX3, EOMES, SMAD2, TGFBR, CXCR4 and Tyrosine protein-kinase (ITK) were differentially activated in the stable states corresponding to phenotypes with only IL-17F versus both IL-17A and F expressed, whereas BLIMP-1, STAT5A, IL-2R, IL-2, IL-2RA, FOXP3, SMAD2, SATB1 and MINA were differentially activated in the stable states corresponding to phenotypes with only IL-17A versus both IL-17A and F expressed. Furthermore, we observed that IL-12R, BLIMP-1, STAT4, RUNX3, EOMES, NFAT2A, SMAD2, STAT5B, STAT5A, IL-2R, IL-2, SATB1, TGFBR, MINA, IL-2RA, CXCR4, FOXP3 and ITK were differentially activated between the phenotypes expressing solely IL-17A and those expressing solely IL-17F. RORγt and RORA activation did not differ between the input conditions analyzed.

Subsequently, an analysis of the regulatory graph was performed to determine the components that directly activate either IL-17A or IL-17F among the nineteen components previously identified. This led us to identify three TFs, NFAT2A, STAT5A and SMAD2, as candidates to explain the observed differential expression of IL-17A and IL-17F.

### Model simulations predict a joint control of IL-17A by SMAD2, STAT5A and NFAT2A in IL-12 + IL-1β condition

In order to further investigate the mechanisms underlying IL-17A expression, we performed in silico perturbations of the three components, presumably involved in IL-17A versus IL-17F differential expression.

For each single or multiple perturbations, we performed stochastic model simulations with MaBoSS for both IL-12 + IL-1β and proTh17 (IL-1β + IL-6 + IL-23 + TGF-β) input conditions, starting from Th0 initial state.

**Fig. 6:**
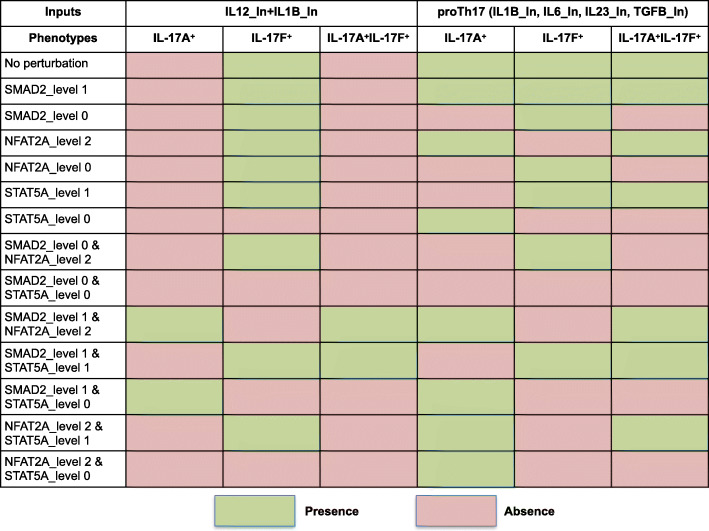
Computational perturbations point to model internal components controlling IL-17A activation. The schema summarizes the impact of selected perturbations (knock-in or knock-out) on IL-12 + IL-1β and Th17 differentiation. Rows denote single or multiple perturbations of SMAD2, STAT5A and NFAT2A. Green and red cells represent activated and inactivated phenotypes, respectively

Figure 6 recapitulates the results of these simulations. In the IL-12 + IL-1β condition, single perturbations of SMAD2, STAT5A or NFAT2A were not sufficient to induce the activation of IL-17A^+^ phenotype. However, an ectopic activation of SMAD2 resulted in the activation of IL-17A^+^IFN-γ^+^IL-17F^+^ phenotype, expressing three cytokines together, while STAT5A knock-out prevented the activation of IL-17A and IL-17F, but enabled the activation of IFN-γ (see Supplementary Table 5 for the IFN-γ phenotypes).

Interestingly, combining an ectopic activation of SMAD2 with either a high ectopic activation of NFAT2A or with a STAT5A knock-out resulted in IL-17A^+^ and IL-17A^+^IL-IL-17F^+^ phenotypes in IL-12 + IL-1β condition. These results suggested an interplay between SMAD2, NFAT2A and STAT5A in the control of IL-17A expression. Furthermore, in the two aforementioned computational perturbation conditions, the IL-17F^+^ phenotype was lost, but this could be restored when SMAD2 or NFAT2A were down-regulated, or when STAT5A is moderately activated. This suggested that IL-17F transcription presumably requires STAT5A, but not SMAD2 nor NFAT2A, and that IL-17A^+^ versus IL-17F^+^ phenotypes could be somehow reversed by modulating STAT5A or NFAT2A when SMAD2 is ectopically activated (see Supplementary Table 5).

The situation was different in the proTh17 condition where the simulation of an ectopic activation of SMAD2, or of a high ectopic activation of NFAT2A, or of a STAT5A knock-out were enough to maintain a stable state characterized by the activation of the sole IL-17A. Finally, in both input conditions, this stable state was lost upon down-regulation of SMAD2 (knock-out) in a NFAT2A ectopic activation or a STAT5A moderate ectopic activation context, pointing towards an essential role of SMAD2 in the induction of IL-17A^+^ phenotypes.

### Integrative analysis: STAT5A is involved in a circuit affecting BLIMP-1, while SMAD2 and NFAT2A are direct activators of IL-17A

In order to explore the mechanisms involving SMAD2, NFAT2A and STAT5A resulting in the differential regulation of IL-17A and IL-17F expression, we performed a detailed analysis of the regulatory graph and observed that the activation of STAT5A can lead to the activation of BLIMP-1, which is required for the expression of IL-17F, but not of IL-17A. Furthermore, IL-12 signaling can trigger the activation of STAT5A. Likewise, STAT1 is regulated by IL-12 and is a direct activator of IL-17F. Finally, SMAD2 and NFAT2A both actívate directly IL-17A (Supplementary Fig. 5). These data suggest that the presence of IL-12 induces a high expression of BLIMP-1, leading to IL-17F expression in IL-12 + IL-1β condition, while in Th17 condition, BLIMP-1 is less expressed.

**Fig. 7:**
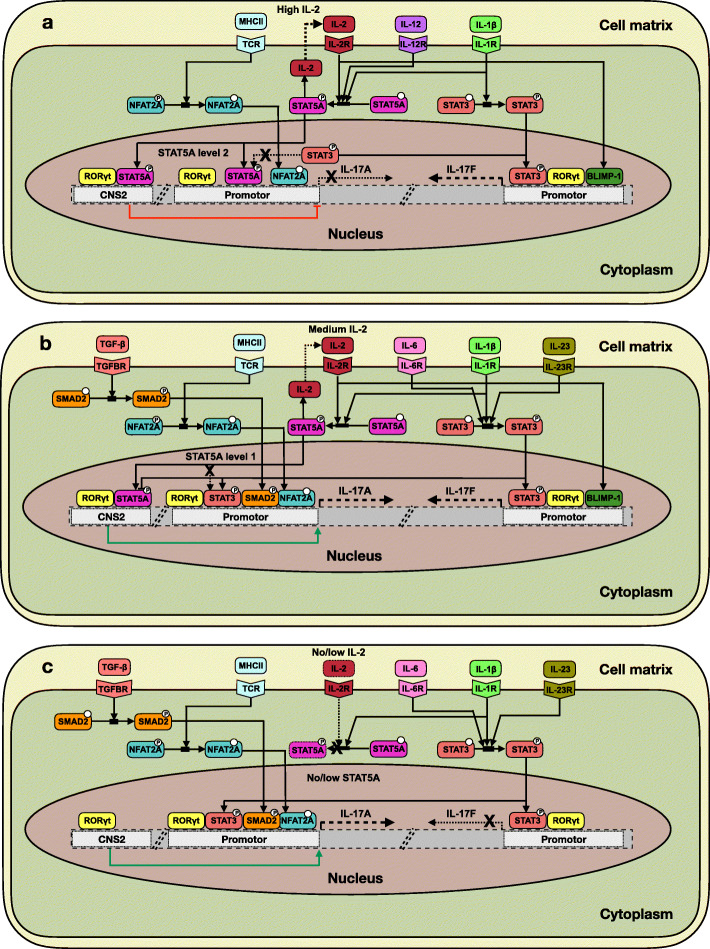
Proposed regulatory mechanism for IL-17A and IL-17F differential regulation. Guided by our modeling study, we further analyzed recent publications to identify mechanisms susceptible to explain the three main cytokine input-output scenarios observed: **a** Expression of IL-17F, but not IL-17A under an IL-12 + IL-1β condition. Note the autocrine loop involving IL-2. The red blunt arc denotes an enhancer-mediated inhibition of IL-17A transcription. **b** Expression of IL-17F and IL-17A in a proTh17 condition. The green arrow denotes an enhancer-mediated activation of IL-17A transcription. **c** Expression of IL-17A, but not IL-17F in a proTh17 condition. The proposed regulatory scheme relies on the following assumptions: i) SMAD2 is required for the expression of IL-17A, ii) SMAD2 and NFAT2A over-expressions have a cooperative effect on IL-17A expression, iii) SMAD2 over-expression and STAT5A down-regulation have a cooperative effect on IL-17A expression, iv) IL-17F transcription presumably requires STAT5A, but not SMAD2 nor NFAT2A, v) STAT5A OFF inhibits IL-17F expression and a low level of STAT5A is necessary, since STAT5A is involved in BLIMP-1 activation circuit and BLIMP-1 is required for IL-17F expression but not for IL-17A expression

Then, as our model analysis points towards combinatorial roles of SMAD2, NFAT2 and STAT5A in the differential expression of IL-17A and IL-17F, we further searched for data supporting molecular mechanisms potentially involved in the interplay between these factors (Supplementary Table 6). Based on current data, we proposed the following scenario to explain the differential expression of IL-17A and IL-17F (Fig. 7):

i) In IL-12 + IL-1β condition, RORγt and STAT5A bind to the Conserved Non-Coding Sequence 2 (CNS2) enhancer to initiate chromatin opening near the IL-17A-IL-17F promoters, RORγt having a positive effect and STAT5A having a direct negative effect on the transcription of these cytokines. The expression of RORγt is presumably not sufficient in this condition to achieve an optimal opening of the chromatin at IL-17A promoter. In addition, in the absence of SMAD2, the negative effect of STAT5A (high level) on IL-17A transcription dominates, resulting in the compacting of chromatin around the STAT3 binding site on the IL-17A promoter, thereby impeding IL-17A expression. However, the inhibitory effect of STAT5A on IL-17F does not impede STAT3 to bind to the IL-17F promoter in the absence of SMAD2, thereby enabling IL-17F transcription. In addition, STAT5A indirectly activates BLIMP-1, a positive regulator of IL-17F required for its expression, leading to the induction of IL-17F (Fig. 7a).

(ii) In proTh17 condition, the expression of RORγt is higher, which allows chromatin opening around the promoters of IL-17A and IL-17F, while SMAD2 is expressed. SMAD2 in turn counteracts STAT5A action and further recruits STAT3, which competes with STAT5A for the binding sites, allowing SMAD2-STAT3 binding at IL-17A promoter. IL-17A can then be expressed in the presence of a high level of STAT5A, since SMAD2 and NFAT2A counteract STAT5A negative effect on IL-17A. IL-17F expression can also be achieved in this condition, based on the mechanisms explained in scenario (i) (Fig. 7b).

(iii) In proTh17 condition, the inhibition of IL-17F expression, observed in IL-17A^+^ phenotypes, is presumably induced by a mechanism driven by STAT5A down-regulation. More precisely, STAT5A inhibition impedes IL-2 expression leading to the down-regulation of the autocrine activation of IL-2 receptors. This, in turn, impedes BLIMP-1 expression and thus IL-17F expression (Fig. 7c).

### Validation of the model prediction: SMAD2 and STAT5A interplay presumably promotes IL-17A production in an IL-12 + IL-1β context

**Fig. 8:**
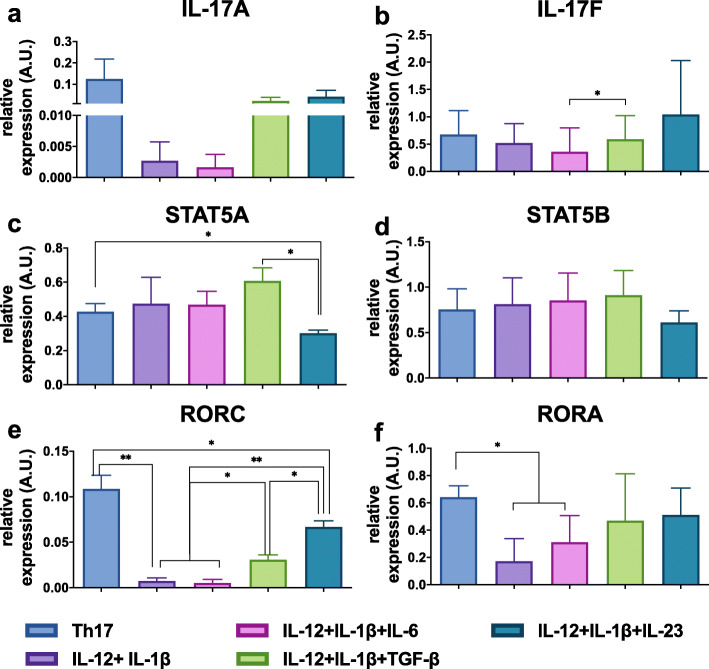
Combinatorial effect of Th17 polarizing cytokines. Human naive T cells were differentiated for 5 days in the presence of polyclonal activation (anti-CD3/anti-CD28 beads). Cells were cultured in the presence of different cytokine inputs: IL-12 + IL-1β, IL-12 + IL-1β + IL-23, IL-1β + IL-23, IL-12 + IL-1β + IL-6, IL-12 + IL-1β + TGF-β, proTh17 (IL-1β + IL-6 + IL-23 + TGF-β). Cells exposed only to polyclonal stimulation were considered as Th0. RNA extraction of differentiated cells was performed and transcripts were then quantified by RT-PCR. Gene expression was normalized to the reference genes HPRT1, B2M and RPL34. **a** Relative expression of IL-17A. **b** Relative expression of IL-17F. **c** Relative expression of STAT5A. **d** Relative expression of STAT5B. **e** Relative expression of RORC. **f** Relative expression of RORA. Graphs represent mean ± SD, N = 3, * and ** represents p < 0.05 and p < 0.01, respectively (paired t-test)

In order to experimentally validate the potential role of SMAD2 in the regulation of IL-17A, we assessed the combinatorial effects of Th17 cytokines on STAT5A, STAT5B, IL-17A and IL-17F expressions by RT-PCR. Upon binding to its receptor, TGF-β can signal through phosphorylation of SMAD2, which translocates to the nucleus to regulate  downstream targets. The increase of SMAD2 proteins has been shown to have a direct effect on STAT3-induced IL-17A production [24]. In our regulatory graph shown in Fig. 2, SMAD2 is activated only by TGF-β. Also, as depicted in Supplementary Fig. 2b, the expression of SMAD2 is significantly increased in the Th17 condition as compared to the IL-12 + IL-1β condition. In our experiments, when TGF-β was added to the IL-12 + IL-1β condition, a slight increase of IL-17A was observed, as compared to the IL-12 + IL-1β condition (Fig. 8a). The expression of STAT5A was also measured in the condition IL-12 + IL-1β + IL-23, where a decrease was observed in comparison with IL-12 + IL-1β + TGF-β and Th17 conditions (Fig. 8c), matching with an increase in IL-17A expression, greater than in IL-12 + IL-1β + TGF-β condition, but not as high as in Th17 condition. RORC expression was almost null in IL-12 + IL-1β condition, while it was moderately expressed in IL-12 + IL-1β + TGF-β and IL-12 + IL-1β + IL-23 condition, suggesting a low requirement of RORC for IL-17F production in the IL-12 + IL-1β condition. On the contrary in Th17 condition, RORC reached its maximum expression (Fig. 8e). RORA expression was different between Th17 and IL-12 + IL-1β conditions (Fig. 8f). The expression of IL-17F and STAT5B were also validated in these proTh17 cytokines-conditions, including IL-12 + IL-1β + TGF-β, where a uniform expression pattern of IL-17F and STAT5B was observed between the conditions (Fig. 8b, d).

BLIMP-1 expression was also validated (Supplementary Fig. 6) and we corroborated that in IL-12 + IL-1β condition the transcription of BLIMP-1 was higher. However, *protein* activation and RNA *transcript* levels do not always perfectly correlate, and the activation level of BLIMP-1 can vary in the same condition, as shown in Supplementary Fig. 4, where BLIMP-1 reached a high level of activation when IL-17A^+^ phenotype was achieved, in a proTh17 condition. These results support the suggested relation between BLIMP-1, STAT5A and IL-17F.

These data corroborates our hypothesis that SMAD2 is essential for the expression of IL-17A in any condition, however a balance in the activity of other TFs, such as STAT5A and RORγt is essential to achieve IL-17A expression. In contrast, RORγt does not seem essential for the IL-17F expression detected in the IL-12 + IL-1β condition. As observed in a Th17 condition, balance between SMAD2, NFAT2A and STAT5A needed to allow the expression of both IL-17A and IL-17F. Altogether, these results point to a positive regulatory role of SMAD2 (activation) and STAT5A (moderate activation) on IL-17A expression.

## Discussion

In this work, logical modeling, experimental calibration and validation strategies were combined to investigate the mechanisms that mediate the differential expression of IL-17A versus IL-17F observed in CD4^+^ T cells.

IL-17A and F differential regulation was previously identified. First, it has been reported that ITK and NFAT2 were involved downstream of TCR signaling in IL-17A activation [17, 18]. Low TCR stimulation would negatively impact IL-17A production rather than IL-17F. Second, PKCα activated by TGBR1 signaling induced IL-17A, but not IL-17F expression [19]. In our study, differential regulation of IL-17A and F could neither be linked to TCR strength, because the same amount of anti-CD3/CD28 were given to cells across conditions, nor to TGF-β signaling alone, because supplementation of TGF-β in the IL-12 + IL-1β condition did not induce IL-17A production. Noteworthy, mouse models poorly recapitulated human mechanistic and phenotypic data regarding Th17 differentiation [25]. Therefore, we rather hypothesized that IL-17A/F differential regulation could be explained by effectors downstream of the IL-12 and IL-1β pathways in T cells.

To address this question, we built a mechanistic logical model of Th17 differentiation integrating the IL-12 and IL-1β pathways together with those usually associated with Th17 differentiation, TGF-β, IL-6 and IL-23. Our model analysis points to a combinatorial role of SMAD2, NFAT2 and STAT5A in the differential expression of IL-17A and IL-17F. Our model was designed on the basis of prior knowledge established by different teams but that was never properly integrated [26, 27].

Our model highlights the role of STAT5A in IL-17A/F differential regulation, together with a cross-talk involving IL-12 and IL-2 pathways, as well as BLIMP-1. Such results were supported by specific knowledge on STAT5A and B proteins regarding different cellular effects arising from differences in the relative amounts of these proteins [28]. In addition, a recent modeling study predicted that the balance between the different STATs defines the amounts of the cytokines produced and thereby T cell phenotypes [29].

During the last decades, IL-12 has been overall the most studied pathway in Th differentiation [20]. This extensive work allowed to discover many regulatory mechanisms associated with IL-12. First, IL-12 was associated with the inhibition of GATA binding protein 3 (GATA-3) and Th2 differentiation [30]. Next, IL-12 was also found to be a positive regulator of BLIMP-1 and B-cell lymphoma 6 protein (BCL-6) in T follicular helper (Tfh) cell differentiation [31]. Several studies reported an inhibitory role of IL-12 on Th17 differentiation [32]. These studies mainly focused on the production of IL-17A and did not look at IL-17F production. Surprisingly, we recently showed that in presence of IL-1β, IL-12 was able to up-regulate IL-17F at very high levels [20]. This led us to consider a differential regulation between IL-17A and IL-17F involving the IL-12p70 pathway. Our results support a positive role of IL-12 in IL-17F induction and therefore in Th17 differentiation, through IL-2, STAT5A and STAT5B activation [50].

Although our model reproduced current experimental data regarding the distinct regulation of IL-17A and F, further mechanistic validations will be required to determine whether our model is complete or if other factors may play a critical role in IL-17A/F differential regulation. In particular, post-transcriptional regulation, known to be important in Th17 differentiation, are not yet explicitly included in our model. For instance, our model predicted that SATB1 activity is moderately high in a Th17 condition (compared to IL-12 + IL-1β), when an IL-17A phenotype is achieved (Supplementary Fig. 4), but transcriptional expression of SATB1 decreases in Th17 condition (RT-PCR experiments). In our favor, SATB1 phosphorylation by PKC increases DNA binding affinity or SATB1 activation [33], and PKC is an IL-17A inducer.

Note also that SMAD3 was not included in our model, although counteracting effects of SMAD2 and SMAD3 have been involved at the *Il17a* locus, according to their level of phosphorylation, with SMAD2 fostering Th17 differentiation, and SMAD3 presumably inhibiting Th17 differentiation [34]. Likewise, epigenetic mechanisms were not considered in our work, although they play a fundamental role in CD4^+^ T cell differentiation. For instance, IL-2 stimulation resulted in a reduction of Histone H3 acetylation, tightly associated with open chromatin structure, primarily at the IL-17A promoter, whereas little effect was observed on the IL-17F promoter [35].

Increasing evidence led to consider that Th17 cells played a pathogenic role in many inflammatory and auto-immune disorders. Many of these studies focused on IL-17A, reducing Th17 to only one critical parameter [36, 37]. Here, we showed that Th17 cells differentiated in presence of IL-1β, IL-6, IL-23 and TGF-β were heterogeneous, coproducing or not IL-17A and F. We also showed that IL-12 could promote “Th17F cells” producing only IL-17F but not IL-17A. This is particularly interesting because several studies observed a pathogenic role of IL-17F, but not IL-17A, in KO mouse models [15, 38], or that the two cytokines can synergize [39]. IL-17F polymorphisms were also associated with several pathologies in human genome-wide association studies [40, 41]. This points to IL-17F as a relevant pathogenic disease-driver even when IL-17A is absent from the microenvironment studied. Unfortunately, the focus on IL-17A, driven by the first mouse model data, led to an underappreciated role of IL-17F in physio-pathological settings. In human clinical settings, IL-17A blocking by monoclonal antibody strategies led to a significant improvement in patient care for several inflammatory and autoimmune disease [42, 43]. However an important fraction of patients do not respond to therapy and could benefit from a combination of IL-17A and F blocking. Currently one monoclonal antibody blocking both IL-17A and F is considered for clinical trial with promising preclinical data [44, 45]. However, there is still no therapeutics dedicated only to IL-17F blocking, which may be crucial if IL-17F promotes inflammation in settings where IL-17A is absent.

## Methods

### Experimental methods

#### Purification of naive CD4^+^ T lymphocytes from adult blood

Peripheral blood mononuclear cells were separated by centrifugation on a density gradient (Lymphoprep, Proteogenix) from apheresis blood obtained from healthy donors (Etablissement Français du Sang, Paris). All cells were used after written informed consent from the donors, and in conformity with institutional and national ethical guidelines. Naive CD4^+^ T lymphocytes were then purified by immunomagnetic depletion using the EasySep Human Naive CD4^+^ T Cell Isolation Kit (StemCell Technologies). The purity of Naive CD4^+^ T cells was over 97.5%.

#### Th cell differentiation assay

Naive CD4^+^ T cells were cultured for 5 days in 48-well plates (Falcon) at a density of 8 × 10^4^ cells per well in X-VIVO 15 serum-free medium (Lonza) and anti-CD3/CD28 Dynabeads (Life Technologies) to obtain the medium control condition (Th0 condition), or in combination with either 10 ng/mL IL-12 (Th1 condition), 10 ng/mL IL-1β (IL-1β condition), IL-12 plus IL-1β (IL-12 + IL-1β condition), or a mix of IL-1β, 100 ng/mL IL-23, 1 ng/mL TGF-β and 20 ng/mL IL-6 to obtain Th17 condition (Peprotech) as previously reported [20] (Fig. 1a). After 5 days, cells were harvested and washed extensively before further analysis (intracellular cytokine staining and real-time quantitative RT-PCR).

#### Flow cytometry and intracellular cytokine staining

For cell surface staining, cells were incubated for 20 min in the dark on ice with PE Mouse anti-human CXCR4 (Clone 12G5; Cat#306505; Biolegend). For the detection of cytokines, naive CD4^+^ T cells were stimulated with 100 ng/mL PMA, 500 ng/mL ionomycin and 3 μg/mL Brefeldin A (ThermoFisher) for 5 h. To exclude dead cells, CD4^+^ T cells were stained using the Zombie NiR fixable viability kit, following manufacturer’s instructions (BioLegend). Cells were then fixed and permeabilized using the IC Fix and Permeabilization buffers (ThermoFisher). Intracellular cytokines were exhibited with the corresponding fluorescence-labelled antibodies: Alexa Fluor® 488 Mouse anti-human IL-17A (Clone BL168; Cat# 512308; BioLegend), PE-Cy7 Rat anti-human IL-17F (Clone SHLR17; Cat# 25–7169-42; ThermoFisher Scientific), BV605 Mouse anti-human IFN-γ (Clone B27; Cat# 562974; BD Biosciences), or matched isotype controls and acquired on a LSRII instrument (BD Biosciences). The data were analyzed with Flowjo software V10.1 (TreeStar).

#### Real-time quantitative RT-PCR

Total RNA was extracted by RNeasy Micro kit (Qiagen) and retro-transcribed using Superscript II Reverse Transcriptase (ThermoFisher Scientific) in combination with random primers, Oligo (dT) and dNTP (Promega). Transcripts were then quantified by PCR on a 480 LightCycler Instrument (Roche). Reactions were performed using a RT-PCR Master Mix Plus (Eurogentec) and TaqMan probes. The following probes (Applied Biosystems, ThermoFisher Scientific) were used: TBX21 (Hs00203436_m1), RORC (RORγt) (Hs01076112_m1), RORA (RORα) (Hs00374280_m1), STAT1 (Hs01014002_m1), STAT3 (Hs00374280_m1), STAT5A (Hs00234181_m1), STAT5B (Hs00560026_m1), IFN-γ (Hs00174143_m1), IL-17A (Hs00174383_m1), IL-17F (Hs00369400_m1), SATB1 (Hs00962580_m1), MINA (Hs01031255_m1). For each sample, mRNA abundance was normalized on the mean of three housekeeping genes (HPRT1 (Hs99999909-m1), B2M (Hs99999907-m1) and RPL34 (Hs00241560_m1)).

#### Statistical analysis

A nonparametric two-tailed Wilcoxon test or a Student’s t-test was used for pair-wise comparisons of cytokines or gene expression. *P* values superior to 0.05 were considered statistically significant.

### Dynamical modeling

#### Regulatory graph construction

We built a comprehensive logical model using the software GINsim, version 3.0 alpha [46], relying on preexisting molecular maps and models. GINsim implements the multivalued logical modeling formalism initially introduced by René Thomas [47]. This formalism relies on the delineation of a regulatory graph, where each component (e.g. protein or gene) is represented by a logical node (taking the values 0 or 1, or additional values when justified), and each regulatory interaction between a pair of components is represented by a signed arc (activation, inhibition, or more rarely a dual interaction, i.e. with a sign depending on the regulator level or on the presence of co-regulators).

RNA-seq and ChIP-seq data in unstimulated naive CD4^+^ T cells were used to support several interactions of the regulatory graph. Data visualization was performed using the Integrative Genomics Viewer and bigWig input files. The software bedGraphToBigWig was used for the conversion of the files. A gene was considered affected by a transcription factor if it could be associated with peaks denoting active promoters or enhancers, together with an expression value of at least 1 RPKM (reads per kilobase of transcripts per million mapped).

#### Dynamical model analysis

To obtain a dynamical model, we further assigned a logical rule to each node of the regulatory graph, which determines its activation level according to the levels of its regulators. These logical rules involve literals (component values) combined with the classical logical operators AND, OR, and NOT [46].

We are particularly interested in the stable states of such models, as they typically represent cellular phenotypes. We computed the stable states of our model using an algorithm implemented in GINsim [46]. In order to estimate the reachability of stable states from relevant initial conditions, we performed stochastic simulations with the software MaBoSS, version 2.0 [48], which translates Boolean networks into continuous time Markov processes. In this framework, each node activation and inactivation is associated with an *up* and a *down rates*, which specify the propensity of the corresponding transitions. From a given state, the simulation integrates all possible node updates and derives a probability and a duration for each transition. For a given set of initial conditions, MaBoSS produces time trajectories and estimates probabilities of model states at each step of the simulation. Steady state distributions can thus be approximated, provided that a sufficient number of sufficiently long simulations have been performed.

All dynamical model analyses, including MaBoSS simulations, have been performed using the CoLoMoTo Docker environment and encoded into a Jupyter Notebook, thereby ensuring the reproducibility of our analyses [49] (cf. Data availability).

#### Model perturbations and hypotheses

Given a logical model, we defined various perturbations to account for experimental observations or to generate predictions regarding the dynamical role of regulatory components. Specifically, we defined single or multiple gene knock-out or knock-in perturbations. The impacts of these perturbations were computed with GINsim and MaBoSS tools. Figure 1b presents our modeling workflow, which encompasses the regulatory graph construction, the simulation and calibration of the model, as well as predictions and validations.

## Supplementary Information

**Additional file 1: Supplementary Figure 1**. Example of Chip-seq data used to infer novel regulations. **Supplementary Fig. 2** Quantification of transcription of selected model components. **Supplementary Fig. 3** The addition of IL-12 to CD4+T cells after three days of differentiation slightly affects the IL-17F+IFN-γ+ cells but not IL-17F+ cells. **Supplementary Fig. 4**. Context-dependent stable state analysis identified key internal model components leading to IL-17A vs IL-17F differential expression. **Supplementary Fig. 5**. STAT5A is involved in a circuit affecting BLIMP-1, while SMAD2 and NFAT2A are direct activators of IL-17A. **Supplementary Fig. 6**. Relative expression of BLIMP-1. **Supplementary Table 1**. Public RNA-seq and Chip-seq data used in this study. **Supplementary Table 2**. Environmental conditions used for the model simulations. **Supplementary Table 3**. Definition of Th subtypes based on the expression of the master regulators and output cytokines. **Supplementary Table 4**. Annotations of the components of the logical model for Th IL-17A and IL-17F differential expression. **Supplementary Table 5**. Impact of selected perturbations on Th cell differentiation in pro Th1 and Th17 conditions. **Supplementary Table 6**. Bibliographical references supporting our tentative mechanistic model for IL-17A and IL-17F differential expression.

## Data Availability

The ChIP-seq datasets used in this study can be downloaded from the database GEO (https://www.ncbi.nlm.nih.gov/geo/), with the identifiers listed in the supplementary Table 1. The logical model can be downloaded from the GINsim model repository at the url: http://ginsim.org/model/ThIL17diff. This file (with a .zginml extension), to be open with the Java software GINsim, version 3.0, freely available at the url http://ginsim.org. The model is also publicly available the database BioModels in the SBML-qual format (https://www.ebi.ac.uk/biomodels/MODEL2101150001).

## Abbreviations

AP-1: Activator protein 1
APC: Antigen presenting cell
BCL-6: B-cell lymphoma 6 protein
BLIMP-1: B lymphocyte-induced maturation protein-1
CD: Cluster of differentiation (CD4 and other CD surface markers)
CNS2: Conserved non-coding sequence 2
CoLoMoTo: Common logical modelling toolbox (software)
CRC6: C-C chemokine receptor type 6
CXCR4: C-X-C motif chemokine receptor 4
EAE: Experimental allergic encephalomyelitis
FOXP3: Forkhead box protein 3
GATA-3: GATA binding protein 3
GINsim: Gene interaction network simulation (software)
IFN: Interferon
IL-17RA: Interleukin-17 receptor A
IL: Interleukin
ITK: Tyrosine protein kinase
KEGG: Encyclopedia of genes and genomes
MHC II: Major histocompatibility complex II
MINA: MYC induced nuclear antigen
MYD88: Myeloid differentiation primary response 88
NF-kB: Nuclear factor kappa B
NFAT: Nuclear factor of activated T cells
PI3K: Phosphoinositide 3-kinases
PKCα: Protein kinase C alpha
RORγt: RAR-related orphan receptor gamma
RT-PCR: Reverse transcription polymerase chain reaction
SATB1: Special AT-rich sequence-binding protein 1
SD: Standard deviation
SMAD: SMAD transcription factor family
STAT: Signal transducer and activator of transcription (TF family)
T-bet: T-box expressed in T cells
TF: Transcription factor
Tfh: T follicular helper (cells)
TGF-β: Transforming growth factor beta
TGFBR1: Transforming growth factor beta receptor 1
Th: T-helper
TL-17RC: Interleukin-17 receptor C
