# Supplementary material for: Interplay between SMAD2 and STAT5A is a critical determinant of IL-17A/IL-17F differential expression
Source: Mol Biomed. 2021 Apr 1;2:9. doi: 10.1186/s43556-021-00034-3 (PMC8607379; doi:10.1186/s43556-021-00034-3)
Supplement: Supplementary file 1 — Additional file 1: Supplementary Figure 1. Example of Chip-seq data used to infer novel regulations. Supplementary Fig. 2 Quantification of transcription of selected model components. Supplementary Fig. 3 The addition of IL-12 to CD4+T cells after three days of differentiation slightly affects the IL-17F+IFN-γ+ cells but not IL-17F+ cells. Supplementary Fig. 4. Context-dependent stable state analysis identified key internal model components leading to IL-17A vs IL-17F differential expression. Supplementary Fig. 5. STAT5A is involved in a circuit affecting BLIMP-1, while SMAD2 and NFAT2A are direct activators of IL-17A. Supplementary Fig. 6. Relative expression of BLIMP-1. Supplementary Table 1. Public RNA-seq and Chip-seq data used in this study. Supplementary Table 2. Environmental conditions used for the model simulations. Supplementary Table 3. Definition of Th subtypes based on the expression of the master regulators and output cytokines. Supplementary Table 4. Annotations of the components of the logical model for Th IL-17A and IL-17F differential expression. Supplementary Table 5. Impact of selected perturbations on Th cell differentiation in pro Th1 and Th17 conditions. Supplementary Table 6. Bibliographical references supporting our tentative mechanistic model for IL-17A and IL-17F differential expression. [file 43556_2021_34_MOESM1_ESM.pdf]

Supplementary Figures and Tables

Interplay between SMAD2 and STAT5A is a critical determinant of IL-17A/IL-17F differential expression.

Karla Fabiola CORRAL-JARA[1], Camille CHAUVIN[2], Wassim ABOU-JAOUDE[1], Maximilien GRANDCLAUDON[3], Aurélien NALDI[1],

Vassili SOUMELIS[2]✳, Denis THIEFFRY[1]✳

[1]Computational Systems Biology Team, Institut de Biologie de l'École Normale Supérieure, CNRS UMR8197, INSERM U1024, École Normale Supérieure, PSL Université, 75005 Paris, France.

[2]Integrative Biology of Human Dendritic Cells and T Cells Team, Institut de Recherche St-Louis, U976, Hôpital Saint Louis, 75010 Paris, France.

[3]Institut Curie, Centre de Recherche, PSL Research University, 75005 Paris, France; INSERM U932, Immunity and Cancer, 75005 Paris, France.

*Corresponding authors.

Email addresses: denis.thieffry@bio.ens.psl.eu, vassili.soumelis@aphp.fr

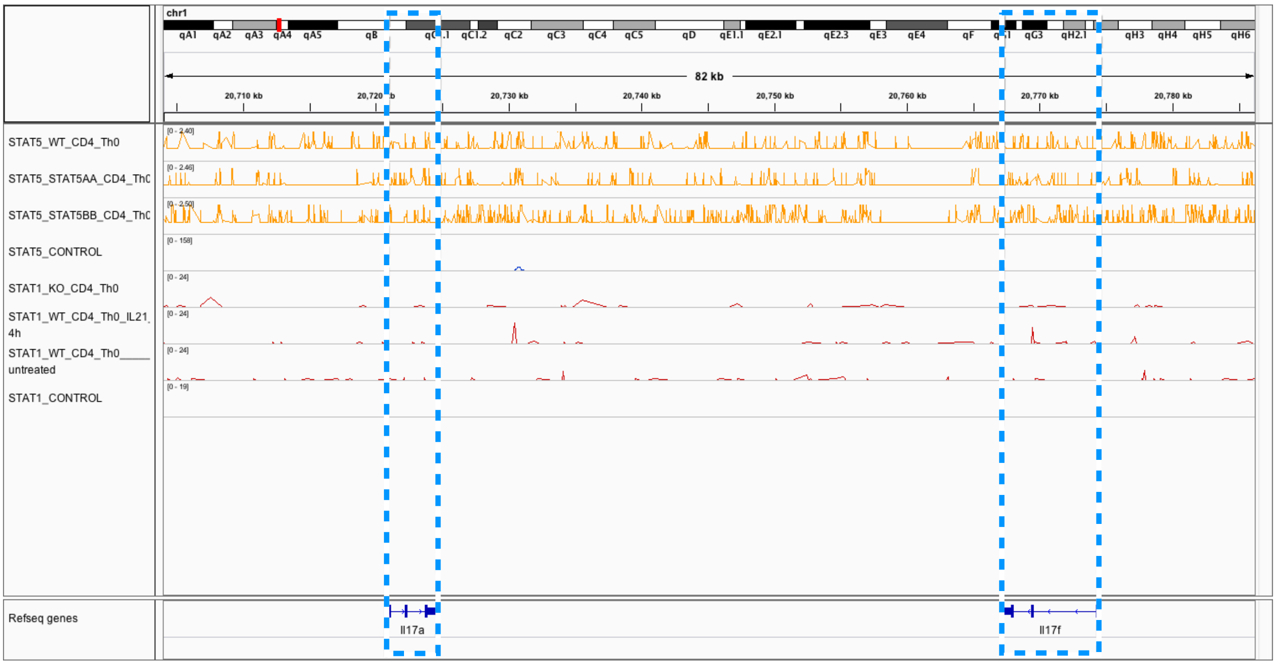

Supplementary Figure 1. Example of Chip-seq data used to infer novel regulations. Integrative Genomics Viewer snapshot displaying Chip-seq peaks for STAT5 and STAT1 in the genomic region encompassing IL-17A and IL-17F genes. Each track corresponds to a ChIP-seq dataset identified by the corresponding GSE ID (Cf. Supplementary Table 1).

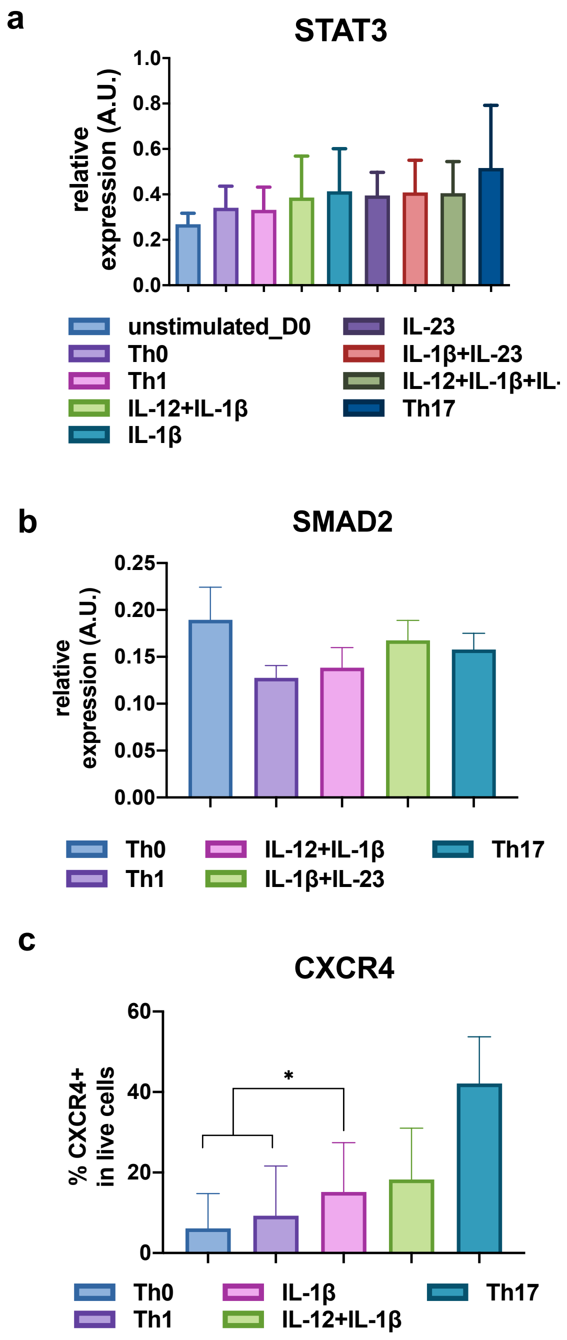

Supplementary Fig. 2 Quantification of transcription of selected model components. Human naive T cells were differentiated for five days in the presence of polyclonal activation (anti-CD3/anti-CD28 beads). Cells were cultured in the presence of different cytokine inputs: proTh1 (IL-12), IL-1β, IL-23, IL-12+IL-1β, IL-1β+IL-23, IL-12+IL-1β+IL-23, proTh17 (IL-1β+ IL-6+ IL-23+TGF-β). Cells exposed only to polyclonal stimulation were considered as Th0. RNA extraction of differentiated cells was performed and transcripts were then quantified by RT-PCR. Gene expression was normalized to the housekeeping genes HPRT1, B2M and RPL34. Relative expressions of STAT3 (N=6) **(a)**, SMAD2 (N=5) **(b).** Expression of CXCR4 was assessed by flow cytometry in **(c)** (N=3). Graphs represent mean ± SD, * denotes $p<0.05$ (Wilcoxon test for panels a and b, paired t-test for panel c).

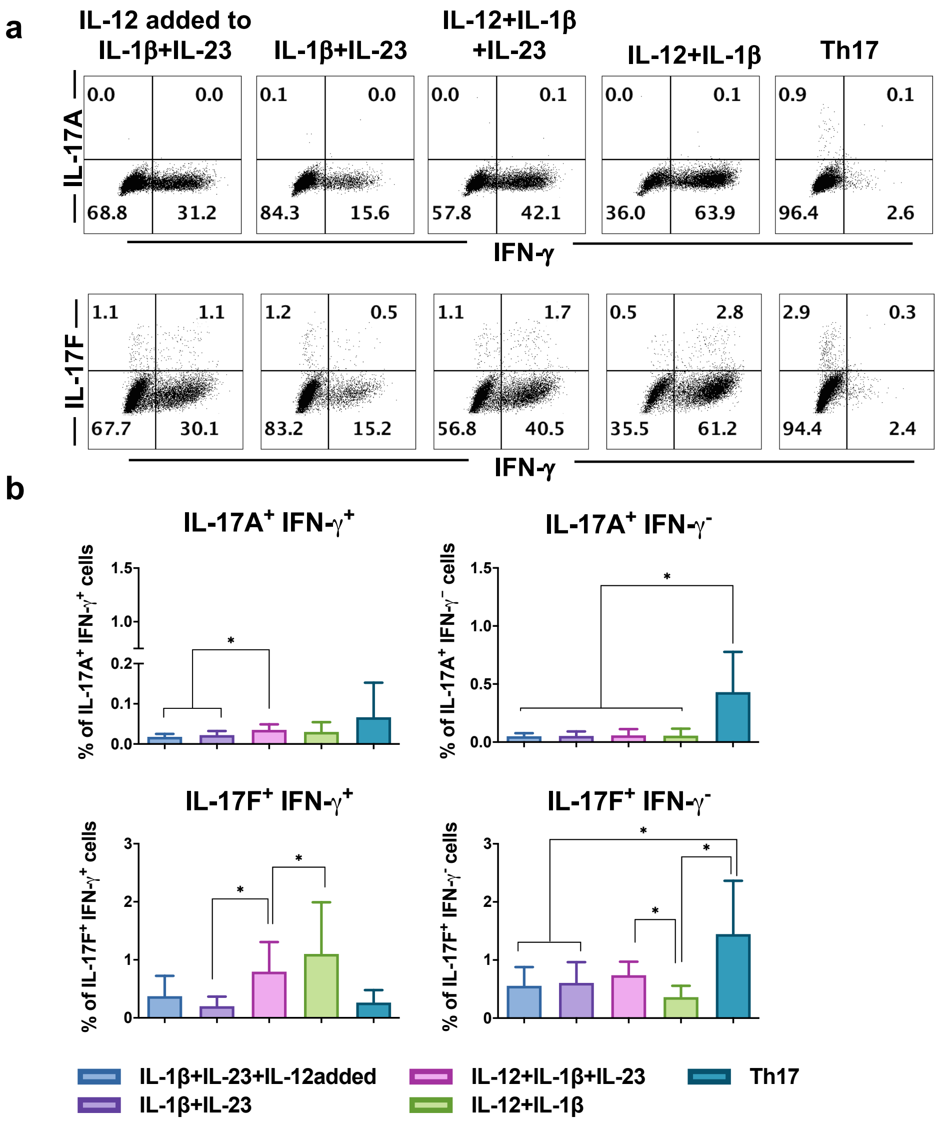

Supplementary Fig. 3 The addition of IL-12 to CD4+T cells after three days of differentiation slightly affects the IL-17F⁺IFN-γ⁺ cells but not IL-17F⁺ cells. Human naive T cells were differentiated for five days in the presence of polyclonal activation (anti-CD3/anti-CD28 beads). Cells were cultured in the presence of different cytokines inputs: IL-12+IL-1β, IL-1β+IL-23, IL-12+IL-1β+IL-23, proTh17 (IL-1β+IL-6+IL-23+TGF-β). After three days, IL-12 was added to cells in the IL-12+IL-1β condition. Differentiated cells were then subjected to flow cytometry analysis. **a)** Dot plot representation of IL-17A and IFN-γ cells in live CD4+ cells is shown in upper panels. Dot plot representation of IL-17F and IFN-γ cells in live CD4+ cells is shown in lower panels. Representative data from two independent experiments are shown. Number denotes frequency of gated cells. **b)** The frequency of cells for each subset in A is shown. Graphs represent mean ± SD, N=6, * denotes $p<0.05$ (Wilcoxon test).

OFF	(red)
LOW	(green)
HIGH	(dark green)

Stable states	Inputs	Phenotypes
**1**	IL12_In+IL-1B_In	IL-17F+
**2**	IL12_In+IL-1B_In	IFN-γ +IL-17F+
**3**	IL6_In+IL1B_In+IL23_In+TGFB_In (Th17)	IL-17A+
**4**	IL6_In+IL1B_In+IL23_In+TGFB_In (Th17)	IL-17A+IL-17F+

	**1**	**2**	**3**	**4**
Tbet	OFF	LOW	LOW	LOW
IL12R	LOW	LOW	OFF	OFF
STAT4	LOW	LOW	OFF	OFF
BLIMP1	HIGH	HIGH	OFF	HIGH
RUNX3	OFF	LOW	LOW	LOW
EOMES	OFF	LOW	LOW	LOW
NFAT2A	LOW	HIGH	HIGH	HIGH
IL2R	HIGH	HIGH	OFF	HIGH
IL2RA	LOW	LOW	OFF	LOW
STAT5B	LOW	LOW	OFF	LOW
IL2	LOW	LOW	OFF	LOW
SMAD2	OFF	OFF	LOW	LOW
STAT5A	HIGH	HIGH	OFF	LOW
FOXP3_2	LOW	LOW	OFF	LOW
SATB1	OFF	OFF	LOW	OFF
MINA	OFF	OFF	LOW	OFF
TGFBR	OFF	OFF	LOW	LOW
CXCR4	OFF	OFF	LOW	LOW
ITK	LOW	LOW	HIGH	HIGH
RORgt	LOW	LOW	LOW	LOW
RORa	LOW	LOW	LOW	LOW
IFNg	OFF	LOW	OFF	OFF
IL17A	OFF	OFF	LOW	LOW
IL17F	LOW	LOW	OFF	LOW

Supplementary Fig. 4. Context-dependent stable state analysis identified key internal model components leading to IL-17A vs IL-17F differential expression. Virtual phenotypes obtained in the following conditions: (i) IL-17F$^+$ and IFN-γ$^+$IL-17F$^+$ cell phenotypes observed in IL-12+IL-1β condition, and (ii) IL-17A$^+$ and IL-17A$^+$IL-17F$^+$ cell phenotypes achieved in proTh17 condition were analyzed. Green cells denote active components (row entries), dark green cells denote the highest activity levels (value 2) in the case of multi-level components and red cells denote inactive components for the corresponding stable state (column entries).

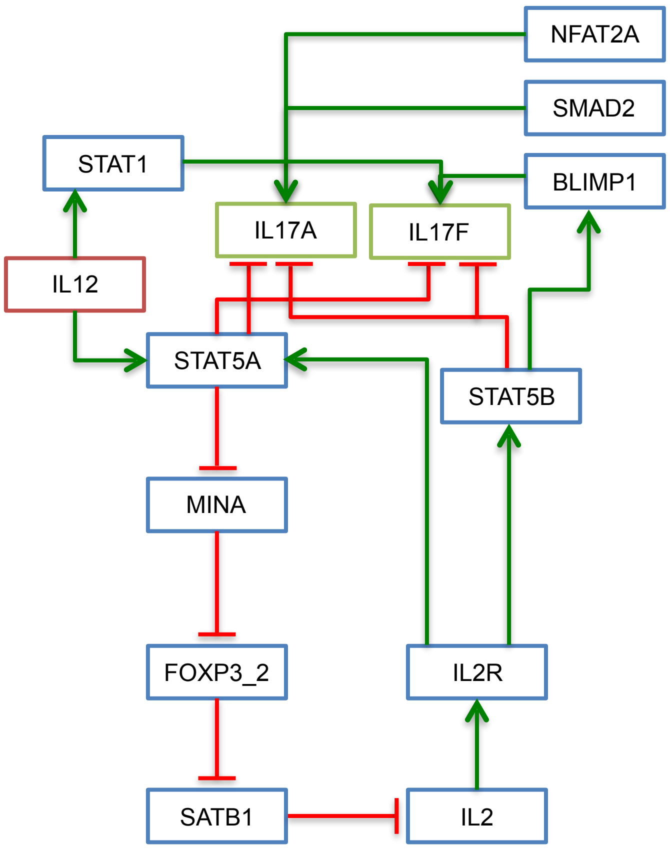

Supplementary Fig. 5. STAT5A is involved in a circuit affecting BLIMP-1, while SMAD2 and NFAT2A are direct activators of IL-17A. Schematic representation of a circuit involving the participation of IL-12, STATs: STAT5A and STAT5B, IL-2 and BLIMP-1. Green arrows and blunt red arcs represent positive and negative interactions, respectively.

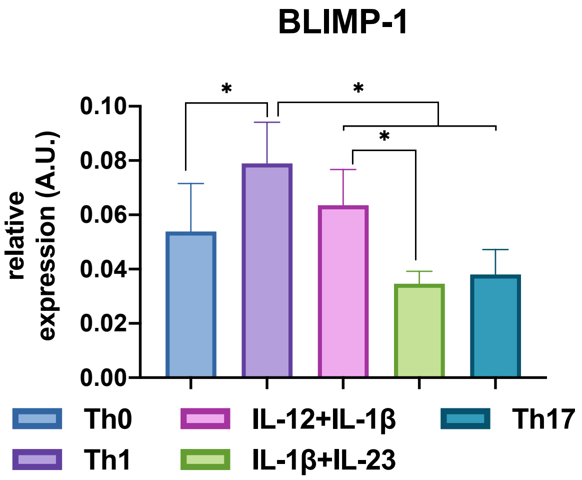

Supplementary Fig. 6. Relative expression of BLIMP-1. Human naive T cells were differentiated for five days in the presence of polyclonal activation (anti-CD3/anti-CD28 beads). Cells were cultured in the presence of different cytokines inputs: IL-12, IL-1β, IL-12+IL-1β, Th2 (IL-4), proTh17 (IL-1β+IL-6+IL-23+TGF-β). Cells exposed only to polyclonal stimulation were considered as Th0. RNA extraction of differentiated cells was performed and transcripts were then quantified by RT-PCR. Gene expression was normalized to the reference genes HPRT1, B2M and RPL34. Graphs represent mean ± SD, N=6 and * denotes $p<0.05$ (Wilcoxon test).

Supplementary Table 1. Public RNA-seq and Chip-seq data used in this study. This table lists the accession IDs of the datasets used to infer the novel interactions integrated in the regulatory graph shown in Fig. 2. All these datasets are gathered in the Gene Expression Omnibus (GEO) database at the NCBI (https://www.ncbi.nlm.nih.gov/geo/).

**Transcription Factor**	**GEO Accesion ID**
STAT3	GSE67183
STAT5A	GSE77656, GSE3954, GSE40869
STAT3	GSE40918
STAT1/STAT3	GSE40463
STAT4	GSE22105
STAT1	GSE63204
STAT1	GSE51531
STAT1/STAT3	GSE40463,GSE60482
STAT1/STAT3	GSE45975

Supplementary Table 2. Environmental conditions used for the model simulations. Each row corresponds to one prototypic environment, defined in terms of combinations of MHCII, CD4, CD80, PIP2, and of five different cytokine inputs: IL-12, IL-1β, IL-23, TGF-β and IL-6.

**Environments**	**Cytokines**	**Model active nodes/inputs**	**Additional active nodes**
proTh0	no cytokines	none	MHCII, CD4, CD80, PIP2
proTh1	IL-12	IL12_In	MHCII, CD4, CD80, PIP2
IL-12+ILβ	IL-12, IL-1β	IL12_In, IL1B_In	MHCII, CD4, CD80, PIP2
IL-1β	IL-1β	IL1B_In	MHCII, CD4, CD80, PIP2
IL-23	IL-23	IL23_In	MHCII, CD4, CD80, PIP2
IL-1β+IL-23	IL-1β, IL-23	IL1B_In, IL23_In	MHCII, CD4, CD80, PIP2
IL-12+IL-1β+IL-23	IL-12, IL-1β, IL-23	IL12_In, IL1B_In, IL12_In	MHCII, CD4, CD80, PIP2
proTh17	IL-1β, IL-23, IL-6, TGF-β	IL1B_In, IL23_In, IL6_In, TGFB_In	MHCII, CD4, CD80, PIP2

Supplementary Table 3. Definition of Th subtypes based on the expression of the master regulators and output cytokines. In the top part, the first row corresponds to the canonical Th cell subtypes expressing no (Th0) or a single master regulator (Th1, Th17). In the bottom part, the rows list the different Th cell subtypes considered, including hybrid subtypes expressing several specific master regulators and proteins. Green cells denote activation and red cells denote inactivation of transcription factors and proteins for each phenotype.

		**Transcription Factors**		**Output Cytokines**		
**Input/ Environment**	**Phenotype description**	**T-bet**	**RORγt**	**IL-17A**	**IL-17F**	**IFN-γ**
Th0	Cells producing no cytokines	Red	Red	Red	Red	Red
Th17	Th17 cell expressing IL-17A and IL-17F	Red	Green	Green	Green	Red
Th1	Th1 cell expressing IFN-γ	Green	Red	Red	Red	Green

		**Transcription Factors**		**Output Cytokines**		
**Phenotype name**	**Phenotype description**	**T-bet**	**RORγt**	**IL-17A**	**IL-17F**	**IFN-γ**
IL-17A	Th17 cell expressing only IL-17A	Red	Green	Green	Red	Red
IL-17F	Th17 expressing only IL-17F	Red	Green	Red	Green	Red
IL-17A/IL-17F	Th17 cell expressing IL-17A and IL-17F	Red	Green	Green	Green	Red
IFN-γ	Th1 cell expressing IFN-γ	Green	Red	Red	Red	Green
IFN-γ/IL-17F	Th1-Th17 hybrid cell expressing IFN-γ and IL-17F	Green	Green	Red	Green	Green
IFN-γ /IL-17A	Th1-Th17 hybrid cell expressing IFN-γ and IL-17A	Green	Green	Green	Red	Green
IFN-γ /IL-17F/ IL-17A	Th1-Th17 hybrid cell expressing IFN-γ , IL-17F and IL-17A	Green	Green	Green	Green	Green

Supplementary Table 4. Annotations of the components of the logical model for Th IL-17A and IL-17F differential expression. For each model component of the model, the left column specifies the corresponding symbol (obeying standard restrictions for computational IDs). The right column provide successively: i) a logical rule combining literals with Boolean operators (cf. Methods) or the mention "input node" when relevant, ii) a short description of the function the node, and iii) supporting references via unique identifiers for specific database entries ("hgnc" refers to HUGO Gene Nomenclature Committee - https://www.genenames.org/, "uniprot" to the reference protein database - https://www.uniprot.org/, and "pmid" to NCBI bibliographical database - https://pubmed.ncbi.nlm.nih.gov/).

**Node ID**	**Value**	**Logical rule** - The symbols !, & and | stand for the Boolean operators NOT, AND and OR, respectively.
**IL1B_In**		Input node, fixed value defined with initial state.
		Interleukin 1 beta [1].<br>External source of IL-1β.
		[1] hgnc:5992
**IL6_In**		Input node, fixed value defined with initial state.
		External source of Interleukin-6.
**IL12_In**		Input node, fixed value defined with initial state.
		Interleukin 12 subunit p40 [1].<br>External source of IL-12.
		[1] hgnc:5970
**IL23_In**		Input node, fixed value defined with initial state.
		Interleukin 23 subunit alpha [1].<br>External source of IL-23.
		[1] hgnc:15488
**TGFB_In**		Input node, fixed value defined with initial state.
		Transforming growth factor beta-1, TGF-β [1].<br>External source of TGF-β .
		[1] uniprot:P01137
**IL6_Aut**	1	PI3K
		Interleukin 6 [1] produced by the cell and further acting autocrinally.<br>A novel role for the phosphatidylinositol 3-kinase/AKT pathway in mediating induction of interleukin-6 (IL-6) in response to IL-1 is reported in [2]. Pharmacological inhibition of phosphatidylinositol 3-kinase (PI3K) inhibited IL-6 mRNA and protein production.
		[1] hgnc:6018
		[2] pmid:18515365
	1	IL12R | IL6R

**STAT1**	STAT1, signal transducer and activator of transcription 1, Transcription factor [1] [2].<br>STAT1 is activated by IL-12, STAT1 is tyrosine phosphorylated in response to IL-12 in PHA-activated human T cells, Figure 1, (functional data in human cells) [3].<br>In STAT3-depleted cells, interleukin IL-6 acquired the capacity to induce apoptosis, correlating with prolonged STAT1 activation and the induction of major histocompatibility complex (MHC) class I expression [4].
	[1] hgnc:11362
	[2] uniprot:P42224
	[3] pmid:9454765
	[4] pmid:19626047
**Tbet**	1 | Tbet | (STAT1 & NFAT1 & AP1 & NFKB & !BLIMP1 & !RUNX1)
	T-bet, T-box 21, TBX21 gene [1].<br>T-bet is the master Transcription Factor of Th1 lineage [2].<br>Auto-activated: Fig 5 (binding and functional data in mice model) [3].<br>Activated by STAT1: Fig 2 (functional data in mice model) [4].<br>NFAT, AP-1 and NF-kβ in combination with STAT1 binds to the TBX21 promoter [5].<br>T-bet is inhibited by BLIMP-1. BLIMP-1 binds directly at multiple sites in TBX21 (binding data in mice model) [6].<br>T-bet is inhibited by RUNX1. Overexpression of RUNX1 was sufficient to reverse the inhibitory effects of T-bet on IL17A production by Th17 cells (functional data in a mice model) [7].<br>In other words, if T-bet is expressed in excess in the cell, it can inhibit the activation of RORc, since it captures RUNX1 and RUNX1 is neccesary for RORc transcription. RUNX1 in adddition to inducing RORc also induces RORa. T-bet suppresses IL-21, which is an important inducer of IL-23. Subsequently, the over-expression of RUNX1 retracts the effect of T-bet on the inhibition of the Th17 response. Experimentally this was corroborated with experimental data, where it was observed that in the IL-12 + IL-1β condition, T-bet is expressed in excess, while RUNX1 and RORc decrease its expression. In the Th17 condition, T-bet decreases its expression and RUNX1 and RORc can increase its expression. Regarding the expression of IL-23R, no differences were observed between the two conditions, experimentally. This indicates the importance of maintaining a balance of expression between TFs, both negative and positive TFs can interact at the same time but at a certain level of expression.<br>We consider that T-bet autoactivation is not affected by the presence of BLIMP-1 and RUNX1, since it occurs when T-bet is overexpressed in the cell and the level of T-bet is enough high.
	[1] hgnc:11599
	[2] uniprot:Q9UL17
	[3] pmid:23232398
	[4] pmid:12006974
	[5] pmid:20103781
	[6] pmid:18684923

	[7] pmid:21151104	
**IFNg**	1	Tbet & RUNX3 & EOMES & STAT4 & STAT1 & NFAT1 & AP1
	Interferon gamma secreted by the cell [1] [2].<br>T-bet and RUNX3 jointly activate IFN- γ (binding and functional data, mice model) [3].<br>Activated by STAT4 (functional data in mice model; binding data) [5].<br>Activated by NFAT (functional data in mice model) [6].<br>AP-1 and STAT4 form a complex that exhibits a stronger force of INFgamma promoter that free alone (binding data in mice cell line) [7].<br>STAT1 binds to IFN- γ gene and induces its transcription [8].<br>EOMES directly up-regulates IFN- γ expression (functional data in mice model) [9].	
	[1] hgnc:5438	
	[2] uniprot:P01579	
	[3] pmid:17195845	
	[4] pmid:12006974	
	[5] pmid:20969595	
	[6] pmid:11520798	
	[7] pmid:11801649	
	[8] pmid:20103781	
	[9] pmid:19050290	
**IL12RB1**	1	Basal value.
	Interleukin 12 receptor subunit beta 1[1].<br>IL2RB1 is a subchain of the IL-12 and IL-23 receptors [2].<br>We consider the constituvely expression of IL12RB1.	
	[1] hgnc:5971	
	[2] uniprot:P42701	
**IL12RB2**	1	Basal value.
	Interleukin 12 receptor subunit beta 2 [1].<br>IL12RB2 is a subchain of the IL-12 receptor [2].<br>We consider the constitutively expression of IL12RB2.	
	[1] hgnc:5972	
	[2] uniprot:Q99665	
**IL12R**	1	IL12_In & IL12RB1 & IL12RB2
	Activated by its subchain(s) IL12RB1, IL12RB2 and by its associated cytokine(s) (external IL12).	
	1	IL12R

**STAT4**	STAT4, Transcription factor [1] [2].<br>Tyrosine residues on the cytoplasmic segment of the IL-12RB2 chain are involved in STAT4 tyrosine phosphorylation, Figure 3 (functional data in mice and human cells) [3].	
	hgnc:11365	
	uniprot:Q14765	
	pmid:12370372	
**IL1RAP**	1	Basal value.
	Interleukin 1 receptor accessory protein [1].<br>IL1RAP is a subchain of IL-1, IL-33 and IL-36 receptors [2].<br>We consider the constitutively expression of IL1RAP.	
	[1] hgnc:5995	
	[2] uniprot:Q9NPH3	
**IL1R1**	1	Basal value.
	Interleukin 1 receptor type 1[1].<br>IL1R1 is a subchain of IL-1 receptor [2].<br>STAT5A activates IL1R1, prediction of Yosef's model (No experimental validation) [3].<br>Yosef´s model is based on diverse databases including mice, rat and human information like TRED, MSigDB 3.0.	
	[1] hgnc:5993	
	[2] uniprot:P14778	
	[3] pmid:23467089	
**STAT3**	1	(IL1R | IL23R | IL6R) & NFKB & AP1
	STAT3, signal transducer and activator of transcription 3, transcription factor [1] [2].<br>Activated by IL23R: Figure 6A (functional data in Kit225 cells) [3].<br>Activated by IL1R (functional data in mice model) [4].<br>IRAK1, a downstream component of IL1R signaling path is mainly involved in STAT3 activation [5].<br>Activated by AP1 and NF-kβ . PKCO mediated activation of AP-1 and NF-kβ for stimulation of the STAT3 promoter (functional data in mice model) [6].<br>It is activated by IL6-gp130 signaling [7].	
	[1] hgnc:11364	
	[2] uniprot:P40763	
	[3] pmid:12023369	
	[4] pmid:19380824	
	[5] pmid:15465816	
	[6] pmid:22586032	
	[7] pmid:17493959	

**IL21**	1	STAT3 & c_Maf & NFAT1
	IL-21 secreted by the cell [1] [2].<br>NFAT together with STAT3 and c-MAF activates the transcription of IL-21 [3].	
	[1] hgnc:6005	
	[2] uniprot:Q9HBE4	
	[3] pmid:20103781	
**IL17A**	1	RORA & RORGt & NFAT2A & SMAD2 & IRF4 & AHR & !(STAT5A & STAT5B) & AP1 & STAT3
	Interleukin IL-17A [1].<br>The components of the model that affect the activation of IL-17F and IL-17A are very similar, except for RORA and NFAT2A, two transcriptional factors possibly affecting only the expression of IL-17A and not of IL-17F [2].<br>IL-17A is activated by STAT3 (functional data in mice models) [3].<br>It is activated by AHR (functional data in mice model) [4].<br>RORγt in combination with AP-1 and IRF4 binds to the IL-17 promoter region to induce transcription [5].<br>BLIMP-1 co-localizes with RORGt and STAT3 at IL-17A/F loci to enhance their expression. With T cells from the dLNs of mice was demonstrated that BLIMP-1-deficient IL-17 producers were not able to co-express GM-CSF and IFN-γ (functional data in mice model) [6].<br>IL-17A expression is not affected by BLIMP-1 (differential chip-seq peaks patterns in IL-17A vs IL-17F promoters) [6].<br>It has corroborated in RORa knockout mice model that the expression of IL-17A and IL23R decreased, however the expression levels of IL-22, IL-21 and IL-17F mRNA were not affected by RORa deficiency, indicating differential dependency of Th17 cytokines on RORa (functional data in mice model) [7].<br>IL-17A expression is linked to NFAT2 activation (functional data in mice model) [8].<br>We suggest that NFAT2A could be affecting the expression of IL-17A.<br>ChIP-seq data analysis revealed that STAT1 could modulate IL-17F directly, but not IL-17A [6].<br>It is known that STAT5 is an inhibitor of IL-17 (functional data in mice model) [9]. However, there is remaining uncertainty regarding the isoforms of STAT5 acting as an inhibitor of IL-17 and wheither this transcription factor preferentially inhibits IL-17F or IL-17A.<br>Hence, we hypothesized that STAT5A and STAT5B inhibit IL-17A. ChIP-seq data show STAT5 peaks in IL-17F and IL-17F genomic regions.<br>SMAD2 is required for the generation of IL-17A in vivo [10]. Experimentaly, we observe that IL-17A expression reaches a high value only when TGF-β is present (and subsequently SMAD2 activation).	
	[1] hgnc:5981	
	[2] uniprot:Q16552	
	[3] pmid:17404271	
	[4] pmid:18607004	
	[5] pmid:20103781	
	[6] pmid:26750311	

<table>
<tr><td rowspan="4"></td><td colspan="2">[7] pmid:18164222</td></tr>
<tr><td colspan="2">[8] pmid:19818650</td></tr>
<tr><td colspan="2">[9] pmid:21278738</td></tr>
<tr><td colspan="2">[10] pmid:20656683</td></tr>
<tr><td rowspan="10">IL17F</td><td>1</td><td>RORGt & AHR & AP1 & IRF4 & BLIMP1 & NFAT1
& (STAT3 | STAT1) & !(STAT5B & STAT5A)</td></tr>
<tr><td colspan="2">IL-17F secreted by the cell [1] [2].
IL-17F transcription is activated by STAT3 (functional data in mice models) [3].
STAT4 is required for IL-17 production in response to IL-23 plus IL-18 (functional data in mice model) [3].
It is activated by AHR (functional data in mice model) [4] [8].
RORγt in combination with AP-1, NFAT, IRF4 binds to the IL-17 promoter region to induce transcription [5].
BLIMP-1 co-localizes with RORγt and STAT3 at IL-17A/F loci to enhance their expression. With T cells from the dLNs of mice was demonstrated that BLIMP-1-deficient IL-17 producers were not able to co-express GM-CSF and IFN-γ (functional data in mice model) [6].
BLIMP-1 peaks are found in IL-17F promoter region, but not in IL-17A promoter region [6].
Hence, we decided to consider a positive interaction from BLIMP-1 onto IL-17F, but not onto IL-17A.
It is known that STAT5 is an inhibitor of IL-17 (functional data in mice model) [7]. However, there is no information about which of the isoforms of STAT5 act as an inhibitor of IL-17, and weither this transcription factor preferentially inhibits IL-17F or IL-17A. ChIP-seq data revealed STAT5 peaks in IL-17F and IL-17F genomic regions (see the model compagnon article).
ChIP-seq data analysis revealed that STAT1 could modulate IL-17F directly, but not IL-17A [6].</td></tr>
<tr><td colspan="2">[1] hgnc:16404</td></tr>
<tr><td colspan="2">[2] uniprot:Q96PD4</td></tr>
<tr><td colspan="2">[3] pmid:17404271</td></tr>
<tr><td colspan="2">[4] pmid:18607004</td></tr>
<tr><td colspan="2">[5] pmid:20103781</td></tr>
<tr><td colspan="2">[6] pmid:26750311</td></tr>
<tr><td colspan="2">[7] pmid:21278738</td></tr>
<tr><td colspan="2">[8] pmid:25261206</td></tr>
<tr><td></td><td>1</td><td>(STAT3 & RUNX1) | (IRF4 & AP1 & NFAT1 & RUNX1 & !Tbet)</td></tr>
</table>

<table>
<tr><td rowspan="8">RORgt</td><td colspan="2">RORγt, RAR related orphan receptor C, Transcription factor [1] [2].
RUNX1 induces RORc expression (Expression and functional data in mice model) [3].
RORγt is inhibited by T-bet. T-bet interacts with the transcription factor RUNX1 and this interaction blocked RUNX1mediated transactivation of RORc (functional data in mice model) [3]. To simplify it has been decided to draw a direct interaction between T-bet and RORγt.
Activated by STAT3 (functional data in mice model) [4].
The development of inflammatory Th17 cells requires IRF4. IRF4 mediates STAT3 and RORγt expression (functional data in mice model) [5].
NFAT and AP-1 binds to the promoter of RORγt which is the master transcription factor of the Th17 lineage [6].
RORγt is induced by STAT5A (binding data) [7].
We hypothesized that there is a RORγt activation mechanism independent of STAT3, however it requires the presence of IRF4, NFAT1, AP-1, RUNX1 and the no presence of T-bet.</td></tr>
<tr><td colspan="2">[1] hgnc:10260</td></tr>
<tr><td colspan="2">[2] uniprot:P51449</td></tr>
<tr><td colspan="2">[3] pmid:21151104</td></tr>
<tr><td colspan="2">[4] pmid:17363300</td></tr>
<tr><td colspan="2">[5] pmid:17676043</td></tr>
<tr><td colspan="2">[6] pmid:20103781</td></tr>
<tr><td colspan="2">[7] pmid:23467089</td></tr>
<tr><td rowspan="4">TCR</td><td>1</td><td>MHCII:1 & Lck</td></tr>
<tr><td>2</td><td>MHCII:2 & Lck</td></tr>
<tr><td colspan="2">T-cell Receptor.
TCR is a molecule found on the surface of T cells, it is responsible to recognizing fragments of antigen as peptides bound to MHC II molecules [1].
The TCR chains all contain immunoreceptor tyrosine-based activation motifs (ITAMs) which are phosphorylated by the Src kinase leukocyte-specific tyrosine kinase (LCK), it is essential to start the intracellular signaling [1].
A high signal strength of TCR is achieved with a high level of MHCII.
TCR signaling components have been validated in Human CD4+ T cells.</td></tr>
<tr><td colspan="2">[1] pmid:19386893</td></tr>
<tr><td rowspan="2">IL1R</td><td>1</td><td>IL1_In & IL1RAP & IL1R1</td></tr>
<tr><td colspan="2">Activated by its subchain(s) IL1R1, IL1RAP and by its associated cytokine(s) (IL1).</td></tr>
<tr><td>CD28</td><td colspan="2">Cluster of Differentiation (CD) 28 [1].
CD28 is one of the proteins expressed on T cells that provide co-stimulatory signals required for T cell activation and survival. CD80, expressed on antigen-presenting cells interacts with CD28 to initiate the immune signal [2].
Ligand binding of CD28 induces the phosphorylation of tyrosine-containing sequences in its cytoplasmic tail by Scr-family kinases like Lck and subsequently induces its activation [3].</td></tr>
</table>

	[1] hgnc:1653	
	[2] uniprot:P10747	
	[3] pmid:19386893	
**LCK**	1	CD4 & TCR
	LCK, lymphocyte-specific protein tyrosine kinase [1] [2].<br>It has been proposed that ligand engagement by the TCR activates the Scr kinase LCK, which in turns phosphorylates the TCR [3].<br>A significant proportion of LCK in the cell constitutively associates with the coreceptor CD4. Because CD4 also interacts with MHC molecules, CD4 recruits LCK to regions that contain TCR complex, allowing the LCK activation [4].	
	[1] hgnc:6524	
	[2] uniprot:P06239	
	[3] pmid:21190897	
	[4] pmid:19386893	
**CD4**	Input node, fixed value defined with initial state	
	CD4 molecule, Cluster of Differentiation 4 [1].<br>Glycoprotein found on the surface of immune cells such T helper cells. CD4 is a coreceptor of the TCR and assits the latter in communicating with antigen-presenting cells [2].	
	[1] hgnc:1678	
	[2] uniprot:P01730	
**ZAP70**	1	Lck & TCR
	ZAP70, Zeta-chain-associated protein kinase 70 [1].<br>Phosphorylated ITAMS (of TCR) recruit the Syk family kinase ZAP70 via Src-homology-2 SH2-domain interactions [2] [3].<br>LCK activates and phosphorylates ZAP70, after which it can transautophosphorylate and activate other vicinal ZAP70 molecules [4].	
	[1] hgnc:12858	
	[2] uniprot:P43403	
	[3] KEGG:hsa04660	
	[4] pmid:29196709	
**LAT**	1	ZAP70
	Linker of Activated T cells [1]<br>LAT is activated by ZAP70 [2] [3].	
	[1] hgnc:18874	
	[2] KEGG:hsa04660	
	[3] uniprot:O43561	
	1	(ZAP70 & LAT) | CD28

**VAV**		Guanine Nucleotide Exhange Factor [1] ,<br>VAV is activated by ZAP70, the VAV site (Y315) in ZAP70 is critical for antigen receptor-mediated signal transduction [2].<br>CD28 signaling is dependent on VAV/Slp6 complex formation and induces membrane localization of these complexes. CD28 can cooperate with VAV/ SLP-76 adaptors to upregulate interleukin 2/4 transcription independently of TCR ligation [3].<br>LAT induces the recruitment of VAV to LAT complex [4].
		[1] uniprot:P15498
		[2] pmid:8673706
		[3] pmid:11754814
		[4] KEGG:hsa04660
**PI3K**	1	TCR:1 & CD28 & RAS & IL1R
	2	TCR:2 & CD28 & RAS & IL1R
		PI3K, Phosphoinositide 3 kinase class IA<br>Class IA PI3Ks are activated by T cell receptor and TCR signaling downstream components, such as by Ras [1].<br>CD28 provides T-cell costimulation and enhances PI3K activity at the immune synapse [2].<br>We suppose that PI3K has a high level when TCR achieve high strength (level 2 also).<br>Validation in Human CD4+ T cell.<br>A novel role for the phosphatidylinositol 3-kinase/AKT pathway in mediating induction of interleukin-6 (IL-6) in response to IL-1 is reported in [3]. Pharmacological inhibition of phosphatidylinositol 3-kinase (PI3K) inhibited IL-6 mRNA and protein production.
		[1] pmid:12660731
		[2] pmid:18006698
		[3] pmid:18515365
**SOS**	1	LAT | CD28
		Son of Sevenless [1].<br>SOS is recruited by LAT through GRB2-related adapter protein, in other words, the activation of SOS requires of LAT [2].<br>CD28 binds the SOS exchange factor and activates it [3].
		[1] uniprot:Q07889
		[2] KEGG:hsa04660
		[3] pmid:7737275
**PLCG**	1	LAT
		Phospholipase C, gamma 1[1] ,<br>The LAT-Slp76 complex acts as a platform for the recruitment of signaling effectors, one of the most important is PLCG, which interacts directly with both LAT and Slp6 [2].

	[1] hgnc:9065	
	[2] pmid:19386893	
**RAC**	1	VAV
	Rho family of GTPases small signaling G proteins. It is induced by VAV [1].	
	[1] KEGG:hsa04660	
**PIP2**	Input node, fixed value defined with initial state.	
	Phosphatydylinositol-4-5-bisphosphate.<br>PIP2 is a negatively charged lipid that, it has a well-established role in the generation of second messengers in T cells signaling pathways [1]	
	[1] pmid:12681284	
**PTEN**	1	FOXP3
	Phosphatase and tensin homolog [1] ,<br>We suggest that FOXP3 induces the expression and activation of PTEN. During Treg induction, PTEN function is maintained through the stabilization of PTEN mRNA transcription and sustained protein levels [2].	
	[1] hgnc:9588	
	[2] pmid:25855357	
**PIP3**	1	((PI3K:1 & !PTEN) | PI3K:2) & PIP2
	Phosphatidylinositol-3,4,5-triphosphate.<br>PI3K phosphorylates PIP2 to form PIP3 on the inner membrane of the cell, thus initiating the downstream signaling components, in other words, to activate PIP3, the presence of PI3K (PI3K in a high level or PI3K in a low level but without the presence of PTEN) and PIP2 is necessary [1].<br>PTEN inhibits PI3K pathway, PTEN dephosphorylates PIP3 [1].	
	[1] pmid:22905034	
**IP3**	1	PLCG & PIP2
	Inositol triphosphate, diffusible second messenger.<br>PLCG transduces TCR signals by hydrolyzing PIP2 to yield DAG and IP3.<br>In other words, to obtain active IP3 is necessary the presence of PLCG and PIP2 [1].	
	[1] pmid:19386893	
**DAG**	1	PLCG & PIP2
	Diacylglycerol, membrane-associated lipid.<br>PLCG transduces TCR signals by hydrolyzing PIP2 to yield DAG and IP3. In other words, the presence of PLCG and PIP2 is necessary to obtain active DAG [1].	
	[1] pmid:19386893	
	1	DAG | PIP3

<table>
<tr><td rowspan="4">PKCO</td><td colspan="2">Protein Kinase C theta
PKC0 is important for T cell activation, proliferation and cytokine production [1].
It is activated by PIP3 through PDK1 [2].
PKC0 is activated by DAG. Upon the initial receptor stimulation, PKC0 is recruited to plasma membrane via membrane-resident DAG binding to its C1 domain [3].</td></tr>
<tr><td colspan="2">[1] pmid:11956228</td></tr>
<tr><td colspan="2">[2] pmid:22905034</td></tr>
<tr><td colspan="2">[3] pmid:26528291</td></tr>
<tr><td rowspan="3">RAS</td><td>1</td><td>SOS | DAG</td></tr>
<tr><td colspan="2">Small GTPase RAS,
DAG activates RAS through RasGRP (RAS guanyl nucleotide-releasing protein) [1].
RAS can also be activated by SOS, which is recruited to LAT via the adaptor molecule Grb2 [1].</td></tr>
<tr><td colspan="2">[1] pmid:19386893</td></tr>
<tr><td rowspan="6">ERK1_2</td><td>1</td><td>RAS & ITK</td></tr>
<tr><td colspan="2">Extracellular signal-regulated kinases, related with Mitogen-activated protein kinase 1 [1].
RAS is a crucial activator of MAPK signaling pathway. RAS starts activating C-Raf, followed by MEK and subsequently ERK [2] [3].
The ERK pathway is stimulated by the association of active RAS [3].
Expression of the Itk variant in primary murine T cells induced higher ERK activation and increased calcium flux upon TCR stimulation compared with wild-type ITK [4].</td></tr>
<tr><td colspan="2">[1] uniprot:P28482</td></tr>
<tr><td colspan="2">[2] KEGG:hsa04660</td></tr>
<tr><td colspan="2">[3] pmid:19386893</td></tr>
<tr><td colspan="2">[4] pmid:20237289</td></tr>
<tr><td rowspan="5">c_FOS</td><td>1</td><td>ERK1_2</td></tr>
<tr><td colspan="2">c-Fos protein, Fos proto-oncogene, AP-1 transcription factor subunit [1] [2].
It is activated by ERK cascade [3].</td></tr>
<tr><td colspan="2">[1] hgnc:3796</td></tr>
<tr><td colspan="2">[2] uniprot:P01100</td></tr>
<tr><td colspan="2">[3] KEGG:hsa04660</td></tr>
<tr><td rowspan="3">AP1</td><td>1</td><td>c_Fos & c_Jun</td></tr>
<tr><td colspan="2">Activator protein 1, Transcription Factor.
MAPK signaling cascades stimulate AP-1 activity via the upregulation of FOS and JUN transcription and also by direct phosphorylation of the FOS and JUN proteins [1].</td></tr>
<tr><td colspan="2">[1] pmid:19386893</td></tr>
</table>

<table>
<tr><td rowspan="6">MEKK1</td><td>1</td><td>RAS | Rac</td></tr>
<tr><td colspan="2">Mitogen-activated protein kinase kinase kinase 1, gene MAP3K1 [1].
MEKK1 initiates the JNK and p38 signaling pathway [1]. A subgroup of MAPKKKs including the MEKK family proteins activate the MAPKKs proteins that phosphorylate JNK and p38 downstream.
Downstream molecules that are activated by JNK include c-JUN [2].
MEKK1 is activated in response to growth factor stimulation of cells and by expression of activated RAS [3].
MEKK1 is activated by RAC. The JNK and p38 pathways respond to activated RAC in addition to RAS [4].</td></tr>
<tr><td colspan="2">[1] uniprot:Q13233</td></tr>
<tr><td colspan="2">[2] pmid:10702308</td></tr>
<tr><td colspan="2">[3] pmid:7744823</td></tr>
<tr><td colspan="2">[4] pmid:19386893</td></tr>
<tr><td rowspan="7">TAK1</td><td>1</td><td>PKCO | TRAF6</td></tr>
<tr><td colspan="2">Transforming growth factor beta-activated kinase 1 or mitogen-activated protein kinase kinase kinase 7 MAP3K7 [1] [2].
TAK1 is activated by upstream molecules comprising PKC0 [3].
TAK1 is activated by TRAF6 [4].
TAK1 is involved in the IL-1 signaling pathway by activating two kinase cascades; one is a MAPK cascade leading to JNK and c-JUN activation and the other is a kinase cascade composed of NF-kβ -inducing kinase and IkB kinases (IKK in the model), ultimately leading to NF-kβ activation [5].</td></tr>
<tr><td colspan="2">[1] hgnc:6859</td></tr>
<tr><td colspan="2">[2] uniprot:O43318</td></tr>
<tr><td colspan="2">[3] pmid:22941947</td></tr>
<tr><td colspan="2">[4] pmid:15125833</td></tr>
<tr><td colspan="2">[5] pmid:10702308</td></tr>
<tr><td rowspan="6">c_JUN</td><td>1</td><td>MEKK1 | TAK1</td></tr>
<tr><td colspan="2">c-JUN protein, Jun proto-oncogene, AP-1 transcription factor subunit [1] [2]
c-JUN is activated by JNK cascade through MEKK1 or TAK1 upstream signaling [3] [4].</td></tr>
<tr><td colspan="2">[1] hgnc:6204</td></tr>
<tr><td colspan="2">[2] uniprot:P05412</td></tr>
<tr><td colspan="2">[3] KEGG:hsa04660</td></tr>
<tr><td colspan="2">[4] pmid:23028407</td></tr>
<tr><td rowspan="2">NFAT1</td><td>1</td><td>IP3</td></tr>
<tr><td colspan="2">Nuclear factor of activated T cells 1.
Also named NFATp or NFATc2 [1] [2].
The production of IP3 stimulates the opening of Ca2+-permeable ion channels in the endoplasmic reticulum. This drives in NFAT induction or activation [3].</td></tr>
</table>

	[1] hgnc:7776	
	[2] uniprot:Q13469	
	[3] pmid:19386893	
**NFKB**	1	IKK
	NF-kβ, Nuclear factor NF-kappa-B p100 subunit [1].<br>Under resting conditions, NF-kβ is sequestred in the cytoplasm by inhibitor of KB (IkB), phosphorylation of IkB by IKK (IkB kinases complex) leads to the degradation of IkB, allowing to NF-kβ to translocate to the nucleus [2].	
	[1] uniprot:Q00653	
	[2] pmid:19386893	
**IKK**	1	PKCO | TAK1
	IKK, IkB kinases enzyme complex.<br>PKCO induces the activation of several adaptor proteins including IKK [1].<br>IKK may itself be regulated by additional enzymes like TAK1 Ser/Thr kinase [1].	
	[1] pmid:17544292	
**c_MAF**	1	STAT3
	c-MAF, Transcription factor Maf [1].<br>STAT3 binds the c-MAF promoter in CD4T cell to activate c-MAF expression (binding data in mice model) [2].	
	[1] uniprot:O75444	
	[2] pmid:15728480	
**MYD88**	1	IL1R
	MYD88, Myeloid differentiation primary response 88 [1] [2].<br>MYD88 is recruited to the IL1 receptor complex following IL-1 stimulation and acts like an adaptor to bind IRAK, therefore IL1R is a an activator of MYD88 [3].	
	[1] hgnc:7562	
	[2] uniprot:Q99836	
	[3] pmid:9430229	
**IRAK1_4**	1	Myd88
	IRAK1-4, Interleukin1 receptor-associated kinase 1 [1] [2] and 4 [3] [4].<br>MYD88 is recruited to the IL-1 receptor complex following IL-1 stimulation and acts like an adaptor to bind IRAK, therefore MYD88 is an activator of IRAK1-4 [5].	
	[1] hgnc:6112	
	[2] uniprot:P51617	
	[3] hgnc:17967	
	[4] UniProtKB - Q9NWZ3	
	[5] pmid:9430229	

<table>
<tr><td rowspan="5">TRAF6</td><td>1</td><td>IRAK1_4</td></tr>
<tr><td colspan="2">TRAF6, TNF receptor associated factor 6 [1] [2].
Autophosphorylated IRAK1-4 interacts with TRAF6, leading to the activation of TRAF6 (functional data in human cells) [3].</td></tr>
<tr><td colspan="2">[1] hgnc:12036</td></tr>
<tr><td colspan="2">[2] uniprot:Q9Y4K3</td></tr>
<tr><td colspan="2">[3] pmid:18070982</td></tr>
<tr><td rowspan="9">BLIMP1</td><td>1</td><td>STAT4 & STAT5B & !STAT3</td></tr>
<tr><td>2</td><td>STAT3 & STAT5B</td></tr>
<tr><td colspan="2">BLIMP-1, PR domain zinc finger protein 1, Prdm1 gene, [1] [2].
BLIMP-1 expression in Th17 cells is STAT3 Dependent, Figure 1 (mice model) [3].
BLIMP-1 is induced by IL-12 in a STAT4-dependent manner, Figure 4 (mice model) [4].
BLIMP-1 Is upregulated by STAT5 (mice model) [5].
Naive CD4+ T cells stimulated under anti-Th1 conditions express higher levels of BLIMP-1 steady-state mRNA [6]. For this reason, we consider that a high level of BLIMP-1 is achieved when STAT3 and STA5B are present and not STAT4 (a pro-Th1 TFs).</td></tr>
<tr><td colspan="2">[1] hgnc:9346</td></tr>
<tr><td colspan="2">[2] uniprot:O75626</td></tr>
<tr><td colspan="2">[3] pmid:26750311</td></tr>
<tr><td colspan="2">[4] pmid:25073792</td></tr>
<tr><td colspan="2">[5] pmid:22318729</td></tr>
<tr><td colspan="2">[6] pmid:18370921</td></tr>
<tr><td rowspan="5">RUNX3</td><td>1</td><td>Tbet</td></tr>
<tr><td colspan="2">RUNX3, Runt related transcription factor 3 [1] [2].
RUNX3 expression during Th1 differentiation requires T-bet (functional data in mice) [3].</td></tr>
<tr><td colspan="2">[1] hgnc:10473</td></tr>
<tr><td colspan="2">[2] uniprot:Q13761</td></tr>
<tr><td colspan="2">[3] pmid:17195845</td></tr>
<tr><td rowspan="5">EOMES</td><td>1</td><td>RUNX3</td></tr>
<tr><td colspan="2">Eomesdermin [1] [2].
It was suggested that RUNX3 induces IFN-γ production partly through its upregulation of EOMES expression (functional data in mice) [3].</td></tr>
<tr><td colspan="2">[1] hgnc:3372</td></tr>
<tr><td colspan="2">[2] uniprot:O95936</td></tr>
<tr><td colspan="2">[3] pmid:20399120</td></tr>
</table>

<table>
<tr><td rowspan="6">IRF4</td><td>1</td><td>AP1 & NFKB</td></tr>
<tr><td colspan="2">Interferon Regulatory factor 4 [1] [2].
NF-kβ subunits p52 and RelB are transcriptional activators of IRFA. The oncogenic transcription factor IRF4 is regulated by a novel CD30/NF-kβ positive feedback loop in peripheral T-cell lymphoma (functional data in clinical samples) [3].
TCR pathway is the major pathway to induce IRF4 in T cells, we suggest that AP-1 is an important activator of IRF4 [4].</td></tr>
<tr><td colspan="2">[1] hgnc:6119</td></tr>
<tr><td colspan="2">[2] uniprot:Q15306</td></tr>
<tr><td colspan="2">[3] pmid:25833963</td></tr>
<tr><td colspan="2">[4] pmid:24782159</td></tr>
<tr><td rowspan="5">AHR</td><td>1</td><td>STAT3</td></tr>
<tr><td colspan="2">Aryl hydrocarbon receptor, transcription factor [1] [2].
STAT3 regulates basal and cytokine-inducible AHR expression in HepG2 cells (binding data) [3].</td></tr>
<tr><td colspan="2">[1] hgnc:348</td></tr>
<tr><td colspan="2">[2] uniprot:P35869</td></tr>
<tr><td colspan="2">[3] pmid:24127753</td></tr>
<tr><td rowspan="4">MHCII</td><td colspan="2">Input node, fixed value defined with initial state.</td></tr>
<tr><td colspan="2">Major Histocompatibility Complex class II,
MHCII molecules bind peptide fragments derived from pathogens and display them on the cell surface for recognition by the appropriate T cells through TCR. Subsequently, immune response initiates [1].
The two levels of MHCII refer to strength of interaction with peptide/MHC ligand and how it transmits the information. A high or low strength can be achieved by changing the amount or quality of peptide/MHC ligand [2]. We consider a high strength stimulation of MHCII (=2) to induce the activation of the virtual cell.</td></tr>
<tr><td colspan="2">[1] Bookshelf ID:NBK27156</td></tr>
<tr><td colspan="2">[2] pmid:21505216</td></tr>
<tr><td rowspan="4">CD80</td><td colspan="2">Input node, fixed value defined with initial state.</td></tr>
<tr><td colspan="2">Cluster of Differentiation 80 [1].
Protein found on dendritic cells, activated B cells and monocytes and provides a costimulatory signal necessary for T cell activation and survival [2].</td></tr>
<tr><td colspan="2">[1] hgnc:1700</td></tr>
<tr><td colspan="2">[2] uniprot:P33681</td></tr>
<tr><td></td><td>1</td><td>IP3</td></tr>
</table>

**NFAT2**	Nuclear factor of activated T cells 2 [1] [2].<br>NFAT activity is regulated by the concentration of intracellular Ca2+. Increases in intracellular Ca2+ lead to the dephosphorylation and nuclear import of NFAT, it means, its activation [3].<br>The production of IP3 stimulates the opening of Ca2+-permeable ion channels in the endoplasmic reticulum. This drives in NFAT induction or activation [3].	
	[1] hgnc:7775	
	[2] uniprot:O95644	
	[3] pmid:19386893	
**NFAT4**	1	IP3
	NFAT4, Nuclear factor of activated T cells 4 [1] [2]<br>The production of IP3 stimulates the opening of Ca2+-permeable ion channels in the endoplasmic reticulum. This drives in NFAT induction or activation [3].	
	[1] hgnc:7777	
	[2] uniprot:Q12968	
	[3] pmid:19386893	
	1	!FOXP3 & NFAT1 & (NFAT4 | NFAT2) & !NFAT2A:2 & ITK
	2	(!(FOXP3 & NFAT1) & ((NFAT1 & NFAT4 & ITK)<br>| (NFAT1 & NFAT2)) & NFAT2A:2 & ITK)<br>| (!(FOXP3 & NFAT1) & NFAT2A:2 & ITK)

**NFAT2A**	NFAT2A, Nuclear factor of activated T cells 2 isoform alpha.<br>Although the main mode of NFAT regulation is through calcium and calcineurin, NFAT2 is apparently unique in that it is also regulated at the transcriptional level through an autoregulatory loop. The mechanism is isoform specific-only one of the two NFAT2 N-terminal splice variants, NFAT2A, is under the control of an NFAT-dependent inducible promoter.<br>Only when sufficiently high levels of NFAT2 expression are attained would the self-sustaining positive autoregulatory loop function to maintain high levels of NFAT2 expression and NFAT activity (murine models) [1].<br>NFAT1 which is the main isoform in naive cells, act together with constitutively expressed NFAT2 and/or NFAT4 to turn on expression of the inducible isoform of NFAT2A (murine models) [1].<br>NFAT2A is inhibited by the complex NFAT1/FOXP3. FOXP3 competes with NFAT1 for binding to the endogenous of NFAT2 promoter. FOXP3 functions not only suppress the first wave of NFAT-mediated transcriptional responses, but may also affect sustained NFAT-mediated inflammatory gene expression through supression of inducible NFAT2 transcriptio [3].<br>It has been corroborated that NFAT1/FOXP3 interaction might affect NFAT1-driven CD40L expression, although this interaction might affect IL-2, IFN-γ, IL-6 and IL-17 production in response to TCR stimulation (functional and expression data in Jurkat cells) [2].<br>Hypothesis: FOXP3+NFAT1 complex inhibits IL-17A expression, through NFAT2A inhibition since NFAT2A induces IL-17A expression when it reaches a high level. This hypothesis is corroborated by experimental results, where NFAT1, NFAT2 and NFAT4 expression does not present changes between different conditions, but the expression of NFAT2A is unknown.<br>Itk−/− cells show a selective defect in the binding of NFATc1 to the IL-17A promoter despite having an open chromatin conformation. ChIP analyses of WT cells differentiated under Th17 conditions demonstrated a large enrichment of NFATc1 binding to the conserved NFAT binding site in the IL-17A promoter. However, we saw no enrichment of amplification in samples from Itk−/− cells [4]. This data suggest that an edge between ITK and NFAT2A should be added to indicates that ITK possibly activates NFAT2 and the latter in turn binds to the IL-17A promoter.	
	[1] pmid:15928679	
	[2] pmid:26324768	
	[3] pmid:19564342	
	[4] pmid:19818650	
	1	FOXP3 | (STAT5B & NFAT1 & SMAD2 & !STAT1 & !RORGt)

<table>
<tr><td rowspan="8">FOXP3</td><td colspan="2">Forkhead box P3 with exon 7, Transcription Factor [1] [2].
Effector T (Teff) cells can transiently express FOXP3 upon activation. STAT5-signaling cytokine induces FOXP3 upregulation in vitro in activated human T effec cells,(expression data in human cells) [3].
Auto-activates (binding and functional data in mice model) [4].
Activated by NFAT and SMAD3 (binding and functional data in mice cells) [5].
Inhibited by RORγt (binding and functional data in human cells) [6].
We assume that STAT1 inhibits FOXP3 (putative binding data in mice model) [7].
We consider that FOXP3 autoactivation is not affected by the presence of STAT1 and RORGt, since it occurs when FOXP3 is overexpressed in the cell and the level of T-bet is enough high.</td></tr>
<tr><td colspan="2">[1] hgnc:6106</td></tr>
<tr><td colspan="2">[2] uniprot:Q9BZS1</td></tr>
<tr><td colspan="2">[3] pmid:18270368</td></tr>
<tr><td colspan="2">[4] pmid:20072126</td></tr>
<tr><td colspan="2">[5] pmid:18157133</td></tr>
<tr><td colspan="2">[6] pmid:20427770</td></tr>
<tr><td colspan="2">[7] pmid:17298177</td></tr>
<tr><td rowspan="3">IL2R</td><td>1</td><td>CGC & IL2RB & !IL2RA & IL2</td></tr>
<tr><td>2</td><td>CGC & IL2RA & IL2 & IL2RB</td></tr>
<tr><td colspan="2">Receptor for IL-2, made of the subchains IL2RA or IL2RB, and CGC, activated by IL-2.
When the IL2RA chain is present, a higher activation level can be reached.</td></tr>
<tr><td></td><td>1</td><td>((SMAD2 & FOXP3) | STAT5B) & NFKB & NFAT2A & !SATB1</td></tr>
</table>

<table>
<tr><td rowspan="9">IL2RA</td><td colspan="2">Interleukin 2 receptor subunit alpha (CD25) [1].
IL2RA is a subchain of the IL-2 receptor [2].
Activated by NFKB: Analysis of the human IL2RA promoter region revealed a critical element bound by NF-kβ (binding and functional data, human cells) [3].
Activated by NFAT: Two NFAT sites were reported to control IL2RA promoter activity in T cells (functional and binding data in mice; also corroborated in human cells) [4].
An interaction between NFAT2alpha and IL2RA is considered in the model.
In addition, a medium level of NFAT2A (=1) is consider as sufficient to activate IL2RA.
IL2RA expression is induced in cells treated with anti-CD3+ anti-CD28, together with TGF-β, through a region containing binding sites for SMAD3 (functional data in mice model) [5].
Activated by FOXP3. It remains to be determined whether FOXP3 directly or indirectly activates the transcription of the IL2RA gene. We consider a direct interaction [6].
Activated by STAT5: Yosef et al (2013) developed a network of TF-target gene associations from published genomics profiles, and predicted a direct interaction between STAT5B and IL2RA [7].
Hence, we consider that STAT5B is sufficient to induce IL2RA expression (in conjunction with NF-kβ andNFAT2A, and that the presence of SMAD3 and FOXP3 is then not necessary.
In the absence of STAT5B, FOXP3 and SMAD3 can activate IL2RA.
SATB1 is a negative regulator of IL12 and IL2RA. SATB1 directly binds to the upstream regulatory region of IL2Ralpha (binding data, human cells) [8].</td></tr>
<tr><td colspan="2">[1] hgnc:6008</td></tr>
<tr><td colspan="2">[2] uniprot:P01589</td></tr>
<tr><td colspan="2">[3] pmid:2497520</td></tr>
<tr><td colspan="2">[4] pmid:9763616</td></tr>
<tr><td colspan="2">[5] pmid:16087671</td></tr>
<tr><td colspan="2">[6] pmid:16911870</td></tr>
<tr><td colspan="2">[7] pmid:23467089</td></tr>
<tr><td colspan="2">[8] pmid:15713622</td></tr>
<tr><td rowspan="4">IL2RB</td><td>1</td><td>Basal value.</td></tr>
<tr><td colspan="2">Interleukin 2 receptor subunit beta [1].
IL2RB is a subchain of the IL-2 and IL-15 receptors [2].
We consider its expression constitutive.</td></tr>
<tr><td colspan="2">[1] hgnc:6009</td></tr>
<tr><td colspan="2">[2] uniprot:P14784</td></tr>
<tr><td rowspan="2"></td><td>1</td><td>IL2R & !((IL2R & !ERK1_2) | !ERK1_2)</td></tr>
<tr><td>2</td><td>(IL2R & !ERK1_2) | !ERK1_2</td></tr>
</table>

**STAT5B**		Signal transducer and activator of transcription 5B, transcription factor [1] [2].<br>Activated by IL2R: (functional and binding data in human cells) [3].<br>Inhibited by ERK [4].<br>The presence of IL2 receptor at a high level (= 2) enables a high level of STAT5B (= 2) , provided that ERK is not present (otherwise, STAT5B can reach the level 1).<br>A medium level of IL2R (= 1) in the absence of ERK leads to medium levels of STAT5B (= 1).
		[1] hgnc:11367
		[2] uniprot:P51692
		[3] pmid:7479881
		[4] pmid:23080204
**CGC**	1	(basal value)
		Common Chain Gamma (Interleukin 2 receptor subunit gamma) [1].<br>CGC is a subchain shared by the IL-2, IL-4, IL-15 and IL-21 receptors [2].<br>We consider the constitutively expression of CGC.
		[1] hgnc:6010
		[2] uniprot:P31785
**IL2**	1	NFKB & AP1 & NFAT1 & !SATB1 & !FOXP3
		Interleukin 2 secreted by the cell [1] [2].<br>Activated by NF-kβ (binding and functional data in mice model) [3].<br>Activated by NFAT (functional data in mice model) [4].<br>AP-1, NF-kβ, NFAT and AP-1 cooperate to activate IL-2 transcription [5].<br>SATB1 is a negative regulator of IL-12 and IL2Ralpha. SATB1 directly binds to the upstream regulatory region of IL2Ralpha (binding data, human cells) [6].<br>Inhibited by FOXP3 (functional data in mice model) [7].
		[1] hgnc:6001
		[2] uniprot:P60568
		[3] pmid:16275766
		[4] pmid:11163226
		[5] pmid:20103781
		[6] pmid:15713622
		[7] pmid:15790681
	1	TGFBR

**SMAD2**		SMAD 2, SMAD family member 2 [1] [2] - SMAD 3, SMAD family member 3 [3] [4].<br>SMAD2 and SMAD3 are activated by TGF-β [5].<br>functional and binding data in mice model show that SMAD2 and SMAD3 are necessary for the differentiation of Tregs [6] through the activation of FOXP3 and IL2RA. In other hand, SMAD2 is required for IL17 differentiation [6] but SMAD3 inhibits it [7] , this indicates unique targets genes of specific SMADs.<br>We consider these interactions in the model with one unique node for SMAD2 and SMAD3, but in the annotation of IL-17A and FOXP3 we specify the only participation of SMAD2 and SMAD3 respectively.
		[1] hgnc:6768
		[2] uniprot:Q15796
		[3] hgnc:6769
		[4] uniprot:P84022
		[5] KEGG:hsa04350
		[6] pmid:20656683
		[7] pmid:19887374
**STAT5A**	1	((IL2R & IL1R) | (IL2R & IL12R)) & !(IL12R & IL1R & IL2R)
	2	IL2R & IL12R & IL1R
		Signal transducer and activator of transcription 5A, transcription factor [1] [2].<br>Activated by IL2R: (functional and bindind data in human cells) [3].<br>Activated by IL-12 and IL-1β, these interactions are induced from qPCR results showing that the expression of STAT5A was higher with the addition of IL-12 and IL-1β together in the medium, in comparison with IL-1β or IL-12 alone.<br>A high level IL2R (level 2), IL-12 and IL-1β cytokines are necessary to push STAT5A at its highest level.
		[1] hgnc:11366
		[2] uniprot:P42229
		[3] pmid:7479881
**GP130**	1	Basal value.
		Glycoprotein 130 (Interleukin 6 signal transducer pseudogene 1) [1].<br>GP130 is a subunit of IL-6, IL-21, IL-23 and IL-27 receptors [2].<br>We consider the constitutively expression of GP130.
		[1] hgnc:6022
		[2] uniprot:P40189
	1	(IL23_In & GP130 & IL12RB1 & STAT3 & RORGt)<br>| (Myd88 & STAT3 & RORGt)

**IL23R**		IL23R, interleukin 23 receptor [1]<br>Composed of the subchains IL12RB1 and GP130, and activated by IL-23 [2].<br>Activated by RORγt:<br>RORγt presumably contributes to directing the expression of IL23R (functional data, mice model) [3].<br>Residual expression of IL23R, consistently found in the absence of RORγt, may be directed by STAT3. IL23R expression is completely abrogated in STAT3-deficient cells (functional data in mice model) [4].<br>MYD88-deficient Th17 cells show reduced IL23R expression and mTOR activation leading to impaired Th17 cell proliferation, suggesting that MYD88 is essential for inducing the IL23R expression necessary for IL-17 production (functional data, mice model) [5].<br>IL23R can be activated by IL-23 input or from internal cellular components, like MYD88.
		[1] hgnc:19100
		[2] uniprot:Q5VWK5
		[3] pmid:17581537
		[4] pmid:17277312
		[5] pmid:23341605
**FOXP3_2**	1	IL1R & !MINA
		FOXP3 forkhead box P3 isoform, excision of exon 7.<br>IL-1β promotes excision of FOXP3 exon7, Figure 2 (human cells and biopsies) [1]. Although the article directly mentions the participation of IL-1β in the induction of FOXP3 isoform, we suggest that it does so through the receptor.<br>MINA promotes the Th17 program and inhibits the FOXP3 expression, Experimental validation in mice models [2].<br>FOXP3 is an inhibitor of SATB1 (see SATB1 annotation).<br>We observed that FOXP3 is expressed in greater quantity in the Th17 condition, while its expression is reduced in the IL-12 + IL-1β condition.<br>SATB1 and MINA are up-regulated in the condition IL-12 + IL-1β and down-regulated in the condition Th17.<br>When MINA is present, FOXP3 lowers its expression and SATB1 is not inhibited by FOXP3, which increases its expression.<br>In the model we hypothesize that FOXP3delta7 is involved in this circuit.
		[1] pmid:26441347
		[2] pmid:23467089
	1	RORGt & !FOXP3_2

**SATB1**		DNA binding protein SATB1[1].<br>FOXP3 acts as a transcriptional repressor, directly supressing the SATB1 locus and indirectly through induction of miRNAs that bound the SATB1 3´UTR (binding data, human cells) [2]. It remains unclear which isoform of FOXP3 inhibits SATB1.<br>SATB1 is induced by RORγt [3]. Yosef's model does not specify whether the interaction is positive or negative and we hypothesize that it is positive based on TF expression data (qPCR measurements).<br>We observe that FOXP3 is expressed in greater quantity in the Th17 condition, while its expression is reduced in the IL-12 + IL-1β condition.<br>SATB1 and MINA are upregulated in the condition IL-12 + IL-1β and downregulated in the condition Th17.<br>When MINA is present, FOXP3 lowers its expression and SATB1 is not inhibited by FOXP3, which increases its expression.<br>In the model, we hypothesize that the FOXP3delta7 is involved in this circuit.
		[1] uniprot:Q01826
		[2] pmid:21841785
		[3] pmid:23467089
**MINA**	1	RORGt & !STAT5A
		MINA is induced by RORγt (validation using a RORγt-knockout mice model) [1]. MINA is presulably inhibited by STAT5A [1]. Yosef's model does not specify whether the interaction is positive or negative and we hypothesize that it is negative in base of TFs expression data (qPCR data).<br>FOXP3 is expressed in greater quantity in the Th17 condition, while its expression is reduced in the IL-12 + IL-1β condition. SATB1 and MINA are up-regulated in the condition IL-12 + IL-1β and down-regulated in the condition Th17. When MINA is present, FOXP3 lowers its expression and SATB1 is not inhibited by FOXP3, which increases its expression. In the model we hypothesize that the FOXP3delta7 isoform is the one that is involved in the mentioned circuit.
		[1] pmid:23467089
**RUNX1**	1	STAT3 & !RORGt
	2	STAT3 & RORGt
		Runt-related transcription factor 1 [1] [2].<br>RUNX1 is presumably activated by RORγt (binding data) [3].<br>It is presumably activated by STAT3(binding data) [3] .<br>An overexpression of RUNX1 is important to reverse the inhibitory effects of T-bet on IL-17A production by Th17 cells, for this reason, we consider two levels of RUNX1 in the model.
		[1] hgnc:10471
		[2] uniprot:Q01196
		[3] pmid:23467089
	1	IRF4 & STAT3 & RUNX1 & RORGt & IL23R

**RORa**	RAR related Orphan Receptor alpha, transcription factor [1] [2].<br>RORa is highly expressed in Th17 cells in a STAT3-dependent manner, Figure 1c (functional data in mice model) [3].<br>Is activated by IRF4, RUNX1, RORγt, these interactions were predicted in [4] (binding data). We assume that a positive interaction is established between the three components and RORa.<br>Hypothesis: IL23R induces directly the expression of RORa, we assume this hypothesis in based on experimental results (qPCR).	
	[1] hgnc:10258	
	[2] uniprot:P35398	
	[3] pmid:18164222	
	[4] pmid:23467089	
**IL6R**	1	GP130 & (IL6_In | IL6_Aut)
	Interleukin 6 receptor [1].<br>Composed of the subchains IL6R and GP130 and activated by external and autocrine IL6 [2].	
	[1] hgnc:6019	
	[2] uniprot:P08887	
**TGFBR**	1	TGFB_In
	Transforming growth factor beta receptor 1 [1].<br>Activated by its subchain(s) and by external TGF-β [2].	
	[1] hgnc:11772	
	[2] uniprot:P01137	
**CXCR4**	1	!(IL12R & IL1R)
	C-X-C motif chemokine receptor 4, CXCR4 [1] , [2].<br>We hypothesized that CXCR4 is inhibited by IL-12 + IL-1β based on our experimental data.	
	[1] hgnc:2561	
	[2] uniprot:P61073	
**ITK**	1	TCR & !CXCR4
	2	TCR & CXCR4
	Interleukin-2 inducible T cell kinase, ITK [1] [2].<br>ITK participates in the crosstalk between TCR and cytokine signaling that differentially affects the activation of distinct signaling pathways involving mTOR and STAT5 activation downstream of IL-2. ITK is activated by TCR [3].<br>We consider that CXCR4 directly activates Itk based on our experimental data.	
	[1] hgnc:6171	
	[2] uniprot:Q08881	
	[3] pmid:24534190	

Supplementary Table 5. Impact of selected perturbations on Th cell differentiation in pro Th1 and Th17 conditions. Suffices _0, _1 or _2 denote a blockade of the corresponding component to the value 0 (knock-out), 1 or 2 (ectopic activation). Single and double perturbations are considered.

**Perturbations**	**IL-12+IL-1β phenotypes**	**Th17 phenotypes**
None	IFN-γ+IL-17F+<br>IL-17F+<br>IFN-γ+	IL-17A+<br>IL-17F+<br>IL-17A+IL-17F+
SMAD2_1	IFN-γ+IL-17F+<br>IFN-γ+IL-17A+IL-17F+<br>IL-17F+<br>IFN-γ+	IL-17F+<br>IL-17A+IL-17F+<br>IL-17A+
SMAD2_0	IFN-γ+<br>IL-17F+<br>IFN-γ+IL-17F+	IL-17F+
NFAT2A_2	IFN-γ+IL-17F+<br>IFN-γ+<br>IL-17F+	IL-17A+IL-17F+<br>IL-17A+
NFAT2A_0	IFN-γ+<br>IL-17F+<br>IFN-γ+IL-17F+	IL-17F+
STAT5A_1	IFN-γ+IL-17F+<br>IL-17F+	IL-17+<br>IL-17A+IL-17F+
STAT5A_0	IFN-γ	IL-17A+
SMAD2_0_NFAT2A_2	IFN-γ+<br>IL-17F+<br>IFN-γ+IL-17F+	IL-17F+
SMAD2_0_STAT5A_0	IFN-γ+	no phenotype
SMAD2_1_NFAT2A_2	IFN-γ+IL-17A+IL-17F+<br>IFN-γ+IL-17A+<br>IL-17A+IL-17F+<br>IL-17A+	IL-17A+IL-17F+<br>IL-17A+
SMAD2_1_NFAT2A_1	IFN-γ+<br>IL-17F+<br>IFN-γ+IL-17F+	IL-17F+
SMAD2_1_NFAT2A_0	IFN-γ+<br>IL-17F+<br>IFN-γ+IL-17F+	IL-17F+
SMAD2_1_STAT5A_1	IFN-γ+IL-17F+<br>IFN-γ+IL-17A+IL-17F+<br>IL-17F+<br>IL-17A+IL-17F+	IL-17F+<br>IL-17A+IL-17F+
SMAD2_1_STAT5A_0	IFN-γ+<br>IFN-γ+IL-17A+<br>IL-17A+	IL-17A+
NFAT2A_2_STAT5A_1	IFN-γ+IL17F+<br>IL-17F+	IL-17A+IL-17F+<br>IL-17A+
NFAT2A_2_STAT5A_0	IFN-γ+	IL-17A+

Supplementary Table 6. Bibliographical references supporting our tentative mechanistic model for IL-17A and IL-17F differential expression. This table list bibliographic references (1st author (year), and PubMed identifier) providing support for the molecular mechanisms presumably underlying the differential expression between IL-17A and IL-17F, as shown in Fig. 7.

Reported data	Reference	PMID
SMAD2 and STAT3 interactions	Jeong-Hwan et al (2015)	26194464
NFATc1 binding sites in IL-17A promoter	Gomez-Rodriguez et al (2009)	19818650
STAT5 repression towards IL-17F	Capone and Volpe (2020)	32226427
SMAD2 and CBP co-activator interactions	Cocolackis et al (2008)	18024957
STAT5 support Th17 activation	Revu et al (2018)	29514093
STAT5 and STAT3 balance to regulate Th17	Xiang-Ping et al (2011)	21278738
General epigenetic mechanisms	Moore et al (2013)	22781841
Histone acetylation mechanism	Sterner and Berger (2000)	10839822
CBP and its HAT activity	Soutoglou et al (2001)	11296231
Epigenetic mechanims in IL-17A expression	Hammitzsch et al (2015)	26261308
Epigenetic mechanims in IL-17A expression	Adamik et al (2013)	23800789
Epigenetic mechanims in IL-17A expression	Wang et al (2012)	22244845
Epigenetic mechanims of STAT5	Ogawa et al (2014)	24298014
Epigenetic mechanims in RORgt expression	Rutz et al (2015)	27481185
Epigenetic mechanims in RORgt expression	He et al (2017)	29158945
